# Data supporting the life cycle impact assessment and cost evaluation of technical alternatives for providing water and heating services to a suburban development in Gällivare Sweden

**DOI:** 10.1016/j.dib.2018.10.058

**Published:** 2018-10-25

**Authors:** Youen Pericault, Erik Kärrman, Maria Viklander, Annelie Hedström

**Affiliations:** a Department of Civil, Environmental and Natural Resources Engineering, Luleå University of Technology, 97187 Luleå, Sweden; b RISE Research Institutes of Sweden, 11894 Stockholm, Sweden

## Abstract

The article presents input data that were used in Pericault et al. (2018) for life cycle impact assessment and total cost assessment of five technical alternatives for heat and water services provision in a suburban development in Sweden. The data consists of a list of environmental impacts (cumulative exergy demand of energy carriers – CExDe, global warming potential – GWP, abiotic depletion potential of elements – ADPE), costs, amortisation periods, lifetimes and output flows of the system processes composing the alternatives. The data was derived from values collected in lifecycle databases, environmental product declarations, scientific publications and personal communications with companies.

**Specifications table**

**Table:** 

Subject area	Civil and environmental engineering
More specific subject area	Urban water engineering, energy engineering
Type of data	Table
How data was acquired	From literature, LCA databases and personal communications with two companies
Data format	Processed
Experimental factors	Data from the ELCD database were converted into environmental impacts with the “CML baseline” and CED impact assessment methods using the Open LCA software. CED values of all system processes were converted to CExDe values using the factors proposed by Bösch et al. [10].
Experimental features	Each technical alternative for heat and water services provision was decomposed in a list of system processes. Environmental impacts, costs, amortisation periods and lifetimes of these processes were collected from scientific databases and publications, environmental product declarations and personal communications with companies. Output flow of each process was estimated by pre-dimensioning the technical alternatives for the residential area of Repisvaara in Gällivare, Sweden.
Data source location	Environmental data: Europe; Cost data: Sweden.
Data accessibility	The data is in the Supplementary material
Related research article	Y. Pericault, E. Kärrman, M. Viklander, A. Hedström, Expansion of sewer, water and district heating networks in cold climate regions: an integrated sustainability assessment, *Sustainability*. **10**, 2018, 3743, https://doi.org/10.3390/su10103743

**Value of the data**

• Part of the data is not site-specific and can be used for life cycle impact assessment (LCA) and life cycle cost assessment of heating, water and sanitation systems.
• Part of the data is site-specific and can be compared to similar data acquired on heating, water and sanitation systems in another context (e.g. warmer climate, other pipe material)
• The majority of the data is related to system processes for water, sewage and heat transport networks and can be used for comparison with water treatment and heat production processes or to study decentralisation levels.

## 1 Data

The data consists of environmental impacts (CExDe, GWP, ADPE), costs, amortisation periods, lifetimes and output flows concerning the system processes of five alternatives studied in [1] for water supply, sanitation and heating in a residential area in Gällivare, Sweden.

## 2 Experimental design, materials, and methods

### 2.1 Pre-dimensioning

A pre-dimensioning of the five alternatives studied in [1] was performed for the residential area of Repisvaara South II [1] to determine the system components and their main characteristics.

#### 2.1.1 Alternative 1 (gravity sewer and high temperature district heating)

PVC pipes of diameter 200 mm were considered for the gravity sewer mains and lateral connections of multi-storey buildings. For the lateral connection of single family homes, PVC pipes of diameter 160 mm were considered. The estimated length of new gravity pipes was 3.7 km including laterals. Inspection manholes in polypropylene (PP) were considered with a spacing of 100 m. It was also evaluated that a gravity sewer system in Repisvaara South II would require one pumping station and a 500 m long rising main in PE (diameter 200 mm). Pre-dimensioning for the drinking water network consisted of mains in PE of diameter 110 mm as well as lateral connection pipes of 63 mm for multi-storey buildings and 40 mm for single family homes. The length of the drinking water network was assumed equal to that of the gravity sewer network. Drinking water and sewer pipes were assumed to be installed under the road in the same trench, at a depth of 2.4 m for frost protection. Trench dimensions, pipe spacing and backfilling materials were chosen in accordance with the Swedish construction code [2]. Pre-dimensioning of the high temperature district heating system was conducted considering a design heat load of 9 kW per single family home and 4.5 kW per apartment. It was therefore evaluated that sub-stations with capacities of 10 kW for single family homes and 100 kW for multi-storey buildings (20 apartments) would be installed. For the distribution network, pre-insulated dual steel pipes manufactured by Powerpipe AB were selected as they are the most common on the Swedish market. Pipe diameter was determined based on the transmission capacities given for the “double++” series in the manufacturer׳s catalogue [3]. The average pipe diameter over the high temperature network was then calculated and corresponded to a DN32 pipe (nominal diameter 32 mm and outer jacket diameter 200 mm). The estimated length of the network was 3.5 km. The pipes were assumed to be buried with a total coverage depth of 1 m. Concerning trench dimensions and backfilling materials, these were chosen in accordance with the Swedish construction code [2].

#### 2.1.2 Alternative 2 (gravity sewer and low temperature district heating)

The pre-dimensioning was conducted considering the same design heat loads as in alternative 1. The average diameter for supply and return district heating pipes was 58 mm while average diameter of the freeze protection pipe was 29 mm. The length of the sewer, drinking water and district heating network was 3.7 km. The same district heating substations as in Alternative 1 were assumed for single family homes and multi-story buildings.

#### 2.1.3 Alternative 3 (low pressure sewer and low temperature district heating)

A pre-dimensioning of the sewerage system was carried out based on the technical handbook of Skandinavisk Kommunalteknik, one of the main suppliers of low pressure sewer (LPS) systems in Scandinavia. The average pipe diameter through the LPS network was 59 mm and the total network length (mains and lateral) was 3.5 km. The grinder pump station selected for single family homes featured a sump of 220 L and an E-one grinder pump (LPS2000E model from Skandinavisk Kommunalteknik). For multi-storey buildings, the pump station featured 3 sumps of 830 L and 10 E-one grinder pumps (two LPS2000Q and one LPS2000D model).

#### 2.1.4 Alternative 4 (gravity sewer and geothermal heat pumps)

A pre-dimensioning of the geothermal heating systems was undertaken considering a capacity of 10 kW for a 180 m deep borehole [4], [5]. It was estimated that single family homes would be equipped with a 10 kW brine-to-water heat pump and a vertical closed-loop collector installed in a 180 m deep borehole. For multi-storey buildings (20 apartments), it was determined that each building would be equipped with a 100 kW heat pump requiring ten 180 m deep boreholes with collectors (10 kW/borehole).

#### 2.1.5 Alternative 5 (low pressure sewer and geothermal heat pumps)

Components composing alternative 5 were already pre-designed in alternative 1 to 4.

### 2.2 Evaluation of environmental impacts

Each alternative was decomposed in a list of system processes (Tables S2–S6), some of which were further decomposed into basic processes (Table S1). Cumulative Energy Demand (CED), GWP and ADPE values of these basic processes were mainly collected from the European Life Cycle Database (ELCD) and Oekobaudat databases [6], [7] and also from environmental product declarations and scientific publications as presented in Table S1. For data extracted from the ELCD [6], an additional step was performed using the “CML baseline” [8] and CED [9] impact assessment methods of the Open LCA software in order to obtain GWP, ADPE and CED values. For each basic process, CExDe values were derived from CED values by using the exergy factors proposed by Bösch et al. [10] which are based on the work of Szargut [11]. CExDe, GWP and ADPE values of system processes (e.g. production of 1 m of pipe) are presented in Tables S2–S6. These were either derived from the environmental impact of basic processes (e.g. production of 1 kg of PE) using factors from the pre-dimensioning step (e.g. mass of PE per meter of pipe), or collected from databases and literature in the same way as for basic processes. Details concerning the evaluation of environmental impacts of basic and system processes are presented in the column “Data sources and assumptions” of Tables S1–S6.

### 2.3 Evaluation of costs, amortisation periods and lifetimes

Costs, amortisation periods and lifetimes of the system processes were collected from the literature and through personal communications with retailers of system components. Details concerning the collection of these values are presented for each system process in the column “Data sources and assumptions” of Tables S2–S6.

### 2.4 Determination of output flows

Output flow of the system processes was derived from the pre-dimensioning of the alternatives (e.g. length of pipe networks) and the characteristics of Repisvaara South II residential area (e.g. number of residential units) presented in [1].
