# Supplementary material for: Data supporting the life cycle impact assessment and cost evaluation of technical alternatives for providing water and heating services to a suburban development in Gällivare Sweden
Source: Data Brief. 2018 Oct 25;21:1204–8. doi: 10.1016/j.dib.2018.10.058 (PMC6231285; doi:10.1016/j.dib.2018.10.058)
Supplement: Supplementary file 2 — Supplementary material. [file mmc2.docx]

Data supporting the life cycle impact assessment and cost evaluation of technical alternatives for providing water and heating services to a suburban development in Gällivare Sweden

**Supplementary material**

**Authors**: Youen Pericault^1^, Erik Kärrman^2^, Maria Viklander^1^, Annelie Hedström^1^

**Affiliations**: ^1^Department of Civil, Environmental and Natural Resources Engineering, Luleå University of Technology, 97187 Luleå, Sweden.

^2^RISE Research Institutes of Sweden, 118 94 Stockholm, Sweden.

**Contact email**: youen.pericault@ltu.se

**Table S1.** **Environmental impacts of basic processes for provision of water and heating services.**

| **#** | **Basic process (used as input for impact assessment of system processes)** | **Cumulative Exergy Demand**  **CExD** | **Global Warming Potential**  **GWP** | **Abiotic Depletion Potential of Elements**  **ADPE** | **Data sources and assumptions** |
| --- | --- | --- | --- | --- | --- |
|  |  | *MJ* | *KgCO_2_Eq* | *KgSbEq* |  |
| 1 | Excavation of 1m3 of sandy soil with 100kW excavator | 1,93E+01 | 1,30E+00 | 6,66E-08 | Oekobaudat 2016 database (BMUB, 2016). Process 9.01.01 “Bagger 100kW Aushub”. |
| 2 | Transport of 1t of good over 1km by medium weight truck | 1,02E+00 | 6,59E-02 | 4,02E-09 | ELCD 3.2 database (ECJRC, 2012). Process “Lorry transport”. |
| 3 | Transport of 1t of good over 1km by heavy weight truck | 7,80E-01 | 5,00E-02 | 3,04E-09 | ELCD 3.2 database. Process “Articulated lorry transport”. |
| 4 | Transport of 1t of good over 1km with sea cargo | 1,80E-01 | 1,00E-02 | 7,07E-10 | ELCD 3.2 database. Process “Container ship ocean”. |
| 5 | Production of 1m3 of crushed rock 16-32mm | 4,23E+01 | 1,11E+00 | 2,22E-06 | Swedish electricity (basic process #18) is consumed for the crushing process according to (Erlandsson, 2010). The crushed rock material is produced from mining waste which is excluded from ADPE calculations. |
| 6 | Production of 1m3 of crushed rock 0-8mm | 6,03E+01 | 1,58E+00 | 3,16E-06 | Swedish electricity (basic process #18) is consumed for the crushing process according to (Erlandsson, 2010). The crushed rock material is produced from mining waste which is excluded from ADPE calculations. |
| 7 | Production of 1kg of PE | 8,02E+01 | 1,95E+00 | 3,50E-08 | ELCD 3.2 database. Process “Polyethylene high density granulate (PE-HD), production mix, at plant”. |
| 8 | Production of 1kg of PP | 7,73E+01 | 1,98E+00 | 5,30E-08 | ELCD 3.2 database. Process “Polypropylene granulate (PP), production mix, at plant”. |
| 9 | Production of 1kg of copper wire | 1,40E+01 | 7,90E-01 | 2,80E-05 | ELCD 3.2 database. Process “Copper wire, consumption mix, at plant, technology mix, cross section 1 mmy”. |
| 10 | Production of 1kg of steel | 1,38E+01 | 1,56E+00 | -9,60E-06 | ELCD 3.2 database. Process “Steel sections (ILCD), production mix, at plant, blast furnace route / electric arc furnace route, 1 kg”. |
| 11 | Production of 1kg of PVC | 6,20E+01 | 2,71E+00 | 1,70E-05 | ELCD 3.2 database. Process “Polyvinylchloride resin (S-PVC), production mix, at plant, suspension polymerisation”. |
| 12 | Production of 1kg of PU foam | 7,74E+01 | 3,20E+00 | 7,80E-06 | (IBU, 2014a) Environmental Product Declaration of PU insulation boards. |
| 13 | Production of 1kg of XPS | 9,05E+01 | 2,82E+00 | 1,29E-06 | (IBU, 2014b) Environmental Product Declaration of XPS insulation. |
| 14 | Production of 1kg of EPS | 9,46E+01 | 3,13E+00 | 1,49E-06 | (IBU, 2013) Environmental Product Declaration of EPS insulation. |
| 15 | Production of 1kg of unsaturated polyester resin | 6,64E+01 | 2,39E+00 | 0,00E+00 | CExD and GWP from (Patel, 2003). ADPE is neglected since polyester resin is mainly produced from organics. |
| 16 | Production of 1kg of glass fibre | 3,11E+01 | 2,01E+00 | 2,70E-06 | ELCD 3.2 database. Process “Continuous filament glass fibre (assembled rovings), at plant”. |
| 17 | Production of 1kg of calcium carbonate | 7,70E-01 | 4,00E-02 | 1,30E-09 | ELCD 3.2 database. Process “Calcium carbonate > 63 microns, at plant, Production”. |
| 18 | Production of 1 MJ of electricity in Sweden | 2,10E+00 | 5,50E-02 | 1,10E-07 | ELCD 3.2 database. Process “Electricity grid mix, consumption mix, at consumer, AC, 230V se”. |
| 19 | Production of 1 MJ of electricity in Poland | 3,39E+00 | 3,10E-01 | 2,22E-09 | ELCD 3.2 database. Process “Electricity grid mix, consumption mix, at consumer, AC, 230V pl”. |
| 20 | Production of 1MJ of heat from peat in CHP plant | 1,23E+00 | 6,58E-02 | 4,44E-09 | The peat has a field density of 800kg/m^3^ and is extracted with a 100kW excavator (basic process #1 above) from Yrttivaara peatland. Transport to Gällivare CHP plant (60km) is performed with medium weight trucks (basic process #2). Production of 1MJ of heat at the CHP plant consumes 1,08MJ of peat and 0,04MJ of electricity (basic process #18), source: CPM LCA database (SLCC, 2000). The calorific value of peat is 9,9 MJ/kg (Bösch et al., 2007). GWP of the combustion (107,5 gCO_2_eq/MJpeat) is mitigated by avoided emissions from the peatland (-42,2 gCO_2_eq/MJpeat) and the afforestation following the harvest (-7,4 gCO_2_eq/MJpeat). GWP values retrieved from (Vaisanen et al., 2013) for forestry-drained peatlands. |
| 21 | Production of 1MJ of heat from wood chips in CHP plant | 1,61E+00 | 1,13E-02 | 1,46E-08 | Impacts of production and transport of 1MJ of wood chips retrieved from (Hagberg et al., 2009) considering roundwood chips dried using biomass fuels. Production of 1MJ of heat at the CHP plant consumes 1,18MJ of wood chips and 0,05MJ of electricity (basic process #18), source: CPM CLA database (SLCC, 2000). |
| 22 | Production of 1MJ of heat from geothermal district heating plant | 5,25E-01 | 1,38E-02 | 2,75E-08 | Geothermal heat is extracted using heat pumps operating with a coefficient of performance of 4. Swedish electricity mix is used (basic process #18). |

Table S2. Output flow, flow unit, lifetime, amortization period, cost and environmental impacts of the system processes composing alternative 1 (gravity sewer and high temperature district heating).

| **System process** | **Output flow** | **Flow unit** | **Lifetime** | **Amortizati-on period** | **Cost** | **Cumulative Exergy Demand CExD** | **Global Warming Potential GWP** | **Abiotic Depletion Potential of Elements ADPE** | **Data sources and assumptions** | |
| --- | --- | --- | --- | --- | --- | --- | --- | --- | --- | --- |
|  |  |  | *Years* | *Years* | €*/unit* | *MJ/unit* | *KgCO_2_Eq/unit* | *KgSbEq/unit* | **Unitary environmental impacts (CExD, GWP, ADPE)** | **Unitary cost, lifetime and amortization period** |
| Trench to frost-free depth for sanitary pipes: Excavation with a 100kW excavator | 3710 | meter | 125 | 50 | 4,70E+02 | 7,69E+01 | 5,17E+00 | 2,65E-07 | Based on basic process #1 considering 3,98m^3^ of soil. | Cost as provided by “WSP Samhällsbyggnad Norrbotten”, consulting company regularly contracted by the water department of Gällivare municipality (L. Björnfot, personal communication, December 09, 2016). Lifetime is the average survival time for new sewer pipes according to the survival function proposed by (Malm et al., 2013).The amortization period is collected from (Nacka, 2012) and is typical for Swedish water utilities.  ↑ |
| Trench to frost-free depth for sanitary pipes: Backfilling with a 100kW excavator | 3710 | meter | 125 | 50 |  | 7,63E+01 | 5,13E+00 | 2,63E-07 | Based on basic process #1 considering 3,95m^3^ of soil. |  |
| Crushed rock 0-8mm: Production | 3710 | meter | 125 | 50 |  | 7,78E+01 | 2,04E+00 | 4,08E-06 | Based on basic process #6 considering 1,29m^3^ of 0-8mm crushed rocks. |  |
| Crushed rock 0-8mm: Transport to construction site | 3710 | meter | 125 | 50 |  | 2,95E+01 | 1,91E+00 | 1,16E-07 | Based on basic process #2 considering 1,29m^3^ of 0-8mm crushed rocks at density 1400 kg/m^3.^ Transportation over 16km from LKAB Malmberget mine. |  |
| Crushed rock 16-32mm: Production | 3710 | meter | 125 | 50 |  | 6,73E+01 | 1,76E+00 | 3,53E-06 | Based on basic process #5 considering 1,59m^3^ of 16-32mm crushed rocks. |  |
| Crushed rock 16-32mm: Transport to construction site | 3710 | meter | 125 | 50 |  | 3,63E+01 | 2,35E+00 | 2,66E-07 | Based on basic process #2 considering 1,59m^3^ of 16-32mm crushed rocks at density 1400 kg/m^3.^ Transportation over 16km from LKAB Malmberget mine. |  |
| Remaining natural soil: Transport to disposal site | 3710 | meter | 125 | 50 |  | 2,37E+01 | 1,54E+00 | 9,36E-08 | Based on basic process #2 considering 2,91m^3^ of natural soil at density 1600 kg/m^3.^ Transportation over 5km to disposal site. |  |
| Gravity sewer pipe in PVC: Production | 3710 | meter | 125 | 50 |  | 2,35E+02 | 1,03E+01 | 6,45E-05 | Based on basic process #11 considering 3,8kg of PVC. Only the production of raw material is considered as it represents most of the environmental load of PVC sewer pipes manufacturing (Carolin et al., 2012). |  |
| Gravity sewer pipe in PVC: Transport to construction site | 3710 | meter | 125 | 50 |  | 1,01E+00 | 6,51E-02 | 3,97E-09 | Based on basic process #2 considering 3,8kg of PVC pipe. Transportation over 260km from Pipelife AB facilities in Haparanda, Sweden. |  |
| Manhole in PP: production | 3710 | meter | 125 | 50 |  | 8,89E+01 | 2,27E+00 | 6,10E-08 | Based on basic process #8 assuming one manhole every 45m of sewer pipe corresponding to 1,15kg of PP per meter of pipe (Carolin et al., 2012). Only the production of raw material is considered. |  |
| Manhole in PP: transport to construction site | 3710 | meter | 125 | 50 | ↑ | 3,05E-01 | 1,97E-02 | 1,20E-09 | Based on basic process #2 considering 1,15kg of PP manhole. Transportation over 260km from Pipelife AB facilities in Haparanda, Sweden. |  |
| Pressurized sewer pipe in PE: Production | 3710 | meter | 125 | 50 |  | 1,32E+02 | 3,22E+00 | 5,77E-08 | Based on basic process #7 considering 1,65kg of PE. Only the production of raw material is considered as it represents most of the environmental load of PE pipes manufacturing (Carolin et al., 2011). |  |
| Pressurized sewer pipe in PE: Transport | 3710 | meter | 125 | 50 |  | 4,37E-01 | 2,83E-02 | 1,72E-09 | Based on basic process #2 considering 1,65kg of PE sewer pipe. Transportation over 260km from Pipelife AB facilities in Haparanda, Sweden. |  |
| Drinking water pipe in PE: Production | 3710 | meter | 125 | 50 |  | 1,59E+02 | 3,88E+00 | 6,96E-08 | Based on basic process #7 considering 1,98kg of PE. Only the production of raw material is considered as it represents most of the environmental load of PE pipes manufacturing (Carolin et al., 2011). |  |
| Drinking water pipe in PE: Transport to construction site | 3710 | meter | 125 | 50 |  | 5,27E-01 | 3,41E-02 | 2,08E-09 | Based on basic process #2 considering 1,98kg of PE pipe. Transportation over 260km from Pipelife AB facilities in Haparanda, Sweden. |  |
| Drinking water pipe in PE: Installation | 3710 | meter | 125 | 50 |  | 0,00E+00 | 0,00E+00 | 0,00E+00 | Environmental load of the installation of the water pipes is neglected. |  |
| Gravity sewer pipe network: Maintenance | 3710 | meter | 1 | - | 4,90E-01 | 0,00E+00 | 0,00E+00 | 0,00E+00 | Environmental load of jetting operations is neglected. | Cost corresponds to the average O&M expenditure for the gravity sewer network of Gällivare (VASS, 2017) during 2013, 2014, 2015 divided by network length. Expenditures related to pumping stations are excluded. |
| Wastewater pumping station: Production and construction | 1 | - | 50 | 50 | 1,15E+05 | 4,28E+04 | 4,81E+03 | -2,93E-02 | 6,5m deep cylindrical excavation of diameter 2,5m for installation of the sump (basic process #1). 3,05 tonnes of stainless steel for the sump and inner wall of the pumping station; the environmental impact of conventional steel is used (basic process #10). Dimensions and mass estimates derived from (Rostfria, 2017). | Cost as provided by the water department of Gällivare municipality (Eliasson, personal communication, December 13, 2016). Lifetime of 50 years is a common value for life cycle analysis of buildings. The amortization period is collected from (Nacka, 2012) and is typical for Swedish water utilities.  ↑ |
| Wastewater pumping station: Transport to construction site | 1 | - | 50 | 50 | ↑ | 7,02E+02 | 4,50E+01 | 2,74E-06 | Based on basic process #3 considering a mass of 3 tonnes^.^ Transportation over 300km from Storfors, Sweden. |  |
| Wastewater pump: Production | 1 | - | 25 | 25 | 2,00E+04 | 9,94E+02 | 1,11E+02 | -6,16E-04 | Based on basic process #10 considering 70kg of steel and on process #9 considering 2kg of copper wire. | Price, Lifetime and amortization period as proposed by (Nacka, 2012). |
| Wastewater pump: Operation and Maintenance | 1 | - | 1 | - | 5,80E+03 | 7,03E+03 | 5,12E+01 | 1,02E-04 | Lifting of wastewater from 100 residential units over 17 meters of elevation. Consumption of electricity (basic process #18) considering a pump efficiency of 55%. Environmental load of maintenance operations is neglected. | Cost corresponds to the average O&M expenditure for sewer pumping stations of Gällivare during 2013, 2014, 2015 divided by the number of pumping station in Gällivare (VASS, 2017). |
| Drinking water distribution network: O&M | 3710 | meter | 1 | - | 1,33E+00 | 6,44E+01 | 4,69E-01 | 9,37E-07 | Derived from O&M costs of drinking water distribution network considering that 50% of cost is due to electrical consumption (basic process #18). | Cost corresponds to the average O&M expenditure for the drinking water distribution network of Gällivare during 2013, 2014, 2015 divided by network length (VASS, 2017). |
| Trench for high temperature district heating pipes: Excavation with a 100kW excavator. | 3510 | meter | 40 | 30 | 1,00E+02 | 9,85E+00 | 6,62E-01 | 3,40E-08 | Based on basic process #1 considering 0,51 m^3^ of soil. | Cost is extracted from Figure 11.6 in (Frederiksen and Werner, 2013) considering a DN32 pipe built in construction site area. Swedish statistics show a considerable increase in failure rate for pre-insulated district heat pipe in steel after 30 years of operation (Åkerström, 2004). Standard for pre-insulated district heating pipes require a minimum lifetime of 50 years (SIS, 2003). The Lifetime used here (40 years) is an average of these two values which is also consistent with the recommendations from the Oekobaudat database (BMUB, 2016) for heating pipes in steel. The amortization of 30 years is selected in accordance with the maintenance handbook of the Swedish district heating association (SDHA, 2015).  ↑ |
| Trench for high temperature district heating pipes: Backfilling with a 100kW excavator. | 3510 | meter | 40 | 30 |  | 9,08E+00 | 6,10E-01 | 3,13E-08 | Based on basic process #1 considering 0,47 m^3^ of soil. |  |
| Crushed rock 0-8mm: Production | 3510 | meter | 40 | 30 |  | 2,71E+01 | 7,11E-01 | 1,42E-06 | Based on basic process #6 considering 0,45 m^3^ of 0-8mm crushed rocks. |  |
| Crushed rock 0-8mm: Transport to construction site | 3510 | meter | 40 | 30 |  | 1,03E+01 | 6,65E-01 | 4,05E-08 | Based on basic process #2 considering 0,45 m^3^ of 0-8mm crushed rocks at density 1400 kg/m^3.^ Transportation over 16km from LKAB Malmberget mine. |  |
| Remaining natural soil: Transport to disposal site | 3510 | meter | 40 | 30 |  | 4,00E+00 | 2,58E-01 | 1,58E-08 | Based on basic process #2 considering 0,49 m^3^ of natural soil at density 1600 kg/m^3.^ Transportation over 5km to disposal site. |  |
| District heating pipes in steel + PU jacket: Production | 3510 | meter | 40 | 30 |  | 2,95E+02 | 1,45E+01 | -4,21E-05 | Production of 5,2kg of steel (basic process #10), 1kg of PU foam (basic process #12) and 1,8kg of PE (basic process #7). |  |
| District heating pipes in steel + PU jacket: Transport to construction site | 3510 | meter | 40 | 30 | ↑ | 9,33E+00 | 5,98E-01 | 3,64E-08 | Based on basic process #3 considering 8kg of district heating pipe. Transportation over 1500km from Powerpipe AB facilities in Gothenburg, Sweden. |  |
| District heating pipes in steel + PU jacket: Installation | 3510 | meter | 40 | 30 |  | 0,00E+00 | 0,00E+00 | 0,00E+00 | Environmental load of district heating pipe installation is neglected. |  |
| District heating pipes in steel + PU jacket: Spot repairs | 3510 | meter | 1 | - | 5,00E-01 | 5,35E-02 | 2,79E-03 | -6,28E-09 | Failure rate of 0,05 km^-1^yr^-1^ is considered for pipes from Powerpipe (Åkerström, 2004). For each failure, excavation and backfilling of 3m of trench (1,41 m^3^ of soil) is considered (basic process #1). Excavator (20 tons) is transported on site with medium weight truck over 5km (basic process #2). | Failure rate of 0,05 km-1yr-1 is considered for pipes from Powerpipe (Åkerström, 2004). Each failure leads to a repair cost of 10000€ (Yarahmadi and Sällström, 2014). |
| High temperature district heating network: Production of heat lost to surrounding soil. | 3510 | meter | 1 | - | 2,18E+00 | 5,00E+02 | 1,17E+01 | 3,45E-06 | Heat losses of 11W/m as provided by the manufacturer (Powerpipe, 2017) for a DN32 pipe "double ++". District heat from Gällivare CHP plant is consumed: 46% peat (basic process #20) and 54% wood chips (basic process #21). Fuel mix is derived from the annual report 2015 of Gällivare energy company (GEAB, 2016). | Heat losses of 11W/m as provided by the manufacturer (Powerpipe, 2017) for a DN32 pipe "double ++". District heat from Gällivare CHP plant is consumed at a marginal cost of 0,02 €/kWh. Marginal cost is derived from the annual report 2015 of Gällivare energy company (GEAB, 2016) by dividing annual O&M costs for heat production (including fuel costs) by annual heat production. |
| District heating substation single family home: Production and installation | 71 | - | 20 | 20 | 1,50E+03 | 8,80E+02 | 6,08E+01 | 2,16E-02 | Based on process 8.1.01 "Substation district heating (A1-A3), 1 kW" from Oekobaudat 2016 database (BMUB, 2016) considering a capacity of 10kW. | Cost as estimated by the Danish Energy Agency (Energistyrelsen, 2012). Lifetime of 20 years is suggested by both the Oekobaudat 2016 database (BMUB, 2016) and the Danish energy agency (Energistyrelsen, 2012). Amortization period of 20 years is suggested by the Swedish district heating association (SDHA, 2007). |
| District heating substation single family home: Transport to construction site | 71 | - | 20 | 20 |  | 1,37E+01 | 8,75E-01 | 5,32E-08 | Substations are transported from Helsinge, Denmark (Perzon et al., 2007) by heavy weight truck (basic process #3). Distance is 1750 km and weight is 10kg (1kg/kW as provided by the Oekobaudat 2016 database (BMUB, 2016)). |  |
| District heating substation single family home: Maintenance | 71 | - | 1 | - | 1,50E+02 | 0,00E+00 | 0,00E+00 | 0,00E+00 | Environmental load of district heating substations maintenance is neglected. | Cost as estimated by the Danish Energy Agency (Energistyrelsen, 2012). |
| District heating substation 20 apartments building: Production | 7 | - | 20 | 20 | 7,00E+03 | 8,80E+03 | 6,08E+02 | 2,16E-01 | Based on process 8.1.01 "Substation district heating (A1-A3), 1 kW" from Oekobaudat 2016 database (BMUB, 2016) considering a capacity of 100kW. | An investment cost of 70€/kW (Energistyrelsen, 2012) is used considering a capacity of 100kW. Lifetime of 20 years is suggested by both the Oekobaudat 2016 database (BMUB, 2016) and the Danish energy agency (Energistyrelsen, 2012). Amortization period of 20 years is suggested by the Swedish district heating association (SDHA, 2007). |
| District heating substation 20 apartments building: Transport to construction site | 7 | - | 20 | 20 | ↑ | 1,37E+02 | 8,75E+00 | 5,32E-07 | Substations are transported from Helsinge, Denmark (Perzon et al., 2007) by heavy weight truck (basic process #3). Distance is 1750 km and weight is 100kg (1kg/kW as provided by the Oekobaudat database (BMUB, 2016)). |  |
| District heating substation 20 apartments building: Maintenance | 7 | - | 1 | - | 5,00E+02 | 0,00E+00 | 0,00E+00 | 0,00E+00 | Environmental load of district heating substations maintenance is neglected. | Cost as estimated by the Danish Energy Agency (Energistyrelsen, 2012). |
| Heat provided to single family home: Production at district heating plant | 71 | - | 1 | - | 3,81E+02 | 8,76E+04 | 2,05E+03 | 6,05E-04 | Annual heat demand of 16900 kWh is considered. District heat from Gällivare CHP plant is consumed: 46% peat (basic process #20) and 54% wood chips (basic process #21). Fuel mix is derived from the annual report 2015 of Gällivare energy company (GEAB, 2016). | Annual heat demand of 16900 kWh is considered. District heat from Gällivare CHP plant is consumed at a marginal cost of 0,02 €/kWh. Marginal cost is derived from the annual report 2015 of Gällivare energy company (GEAB, 2016) by dividing annual O&M costs for heat production (including fuel costs) by annual heat production. |
| Heat delivered to 20 apartments building: Production at district heating plant | 7 | - | 1 | - | 3,63E+03 | 8,35E+05 | 1,95E+04 | 5,77E-03 | Annual heat demand of 161000 kWh is considered (20*8050kWh). District heat from Gällivare CHP plant is consumed: 46% peat (basic process #20) and 54% wood chips (basic process #21). Fuel mix is derived from the annual report 2015 of Gällivare energy company (GEAB, 2016). | Annual heat demand of 161000 kWh is considered (20*8050kWh). District heat from Gällivare CHP plant is consumed at a marginal cost of 0,02 €/kWh. Marginal cost is derived from the annual report 2015 of Gällivare energy company (GEAB, 2016) by dividing annual O&M costs for heat production (including fuel costs) by annual heat production. |

Table S3. Output flow, flow unit, lifetime, amortization period, cost and environmental impacts of the system processes composing alternative 2 (gravity sewer and low temperature district heating).

| **System process** | **Output flow** | **Flow unit** | **Lifetime** | **Amortizati-on period** | **Cost** | **Cumulative Exergy Demand CExD** | **Global Warming Potential GWP** | **Abiotic Depletion Potential of Elements ADPE** | **Data sources and assumptions** | |
| --- | --- | --- | --- | --- | --- | --- | --- | --- | --- | --- |
|  |  |  | *Years* | *Years* | €*/unit* | *MJ/unit* | *KgCO_2_Eq/unit* | *KgSbEq/unit* | **Unitary environmental impacts (CExD, GWP, ADPE)** | **Unitary cost, lifetime and amortization period** |
| Trench for LTDH and gravity sewer: Excavation with a 100kW excavator. | 3710 | meter | 40 | 30 | 7,00E+01 | 1,45E+01 | 9,74E-01 | 4,99E-08 | Based on basic process #1 considering 0,75 m^3^ of soil. | Cost of a shallow trench including installation of water and sewer pipes as provided by the water utility of Kiruna (Skoglind, 2014). Kiruna is a municipality nearby Gällivare with similar labour costs. Lifetime of low temperature district heating pipes in PEX in used. The amortization period of 30 years is selected in accordance with the maintenance handbook of the Swedish district heating association (SDHA, 2015).  ↑ |
| Trench for LTDH and gravity sewer: Backfilling with a 100kW excavator. | 3710 | meter | 40 | 30 |  | 9,66E+00 | 6,49E-01 | 3,33E-08 | Based on basic process #1 considering 0,5 m^3^ of soil. |  |
| Crushed rocks 0-8mm: Production | 3710 | meter | 40 | 30 |  | 2,90E+01 | 7,58E-01 | 1,52E-06 | Based on basic process #6 considering 0,48 m^3^ of 0-8mm crushed rocks. |  |
| Crushed rocks 0-8mm: Transport to construction site | 3710 | meter | 40 | 30 |  | 1,10E+01 | 7,09E-01 | 4,32E-08 | Based on basic process #2 considering 0,48 m^3^ of 0-8mm crushed rocks at density 1400 kg/m^3.^ Transportation over 16km from LKAB Malmberget mine. |  |
| Remaining natural soil: Transport to disposal site | 3710 | meter | 40 | 30 |  | 5,96E+00 | 3,85E-01 | 2,35E-08 | Based on basic process #2 considering 0,73 m^3^ of natural soil at density 1600 kg/m^3.^ Transportation over 5km to disposal site. |  |
| Gravity sewer pipe in PVC: Production | 3710 | meter | 125 | 50 |  | 2,35E+02 | 1,03E+01 | 6,45E-05 | Based on basic process #11 considering 3,8kg of PVC. Only the production of raw material is considered as it represents most of the environmental load of PVC sewer pipes manufacturing (Carolin et al., 2012). |  |
| Gravity sewer pipe in PVC: Transport to construction site | 3710 | meter | 125 | 50 |  | 1,01E+00 | 6,51E-02 | 3,97E-09 | Based on basic process #2 considering 3,8kg of PVC pipe. Transportation over 260km from Pipelife AB facilities in Haparanda, Sweden. |  |
| Manhole in PP: production | 3710 | meter | 125 | 50 |  | 8,89E+01 | 2,27E+00 | 6,10E-08 | Based on basic process #8 assuming one manhole every 45m of sewer pipe corresponding to 1,15kg of PP per meter of pipe (Carolin et al., 2012). Only the production of raw material is considered. |  |
| Manhole in PP: transport to construction site | 3710 | meter | 125 | 50 |  | 3,05E-01 | 1,97E-02 | 1,20E-09 | Based on basic process #2 considering 1,15kg of PP manhole. Transportation over 260km from Pipelife AB facilities in Haparanda, Sweden. |  |
| Pressurized sewer pipe in PE: Production | 3710 | meter | 125 | 50 |  | 1,32E+02 | 3,22E+00 | 5,77E-08 | Based on basic process #7 considering 1,65kg of PE. Only the production of raw material is considered as it represents most of the environmental load of PE pipes manufacturing (Carolin et al., 2011). |  |
| Pressurized sewer pipe in PE: Transport | 3710 | meter | 125 | 50 | ↑ | 4,37E-01 | 2,83E-02 | 1,72E-09 | Based on basic process #2 considering 1,65kg of PE sewer pipe. Transportation over 260km from Pipelife AB facilities in Haparanda, Sweden. |  |
| Drinking water pipe in PE: Production | 3710 | meter | 125 | 50 |  | 1,59E+02 | 3,88E+00 | 6,96E-08 | Based on basic process #7 considering 1,98kg of PE. Only the production of raw material is considered as it represents most of the environmental load of PE pipes manufacturing (Carolin et al., 2011). |  |
| Drinking water pipe in PE: Transport to construction site | 3710 | meter | 125 | 50 |  | 5,27E-01 | 3,41E-02 | 2,08E-09 | Based on basic process #2 considering 1,98kg of PE pipe. Transportation over 260km from Pipelife AB facilities in Haparanda, Sweden. |  |
| Drinking water pipe in PE: Installation | 3710 | meter | 125 | 50 |  | 0,00E+00 | 0,00E+00 | 0,00E+00 | Environmental load of the installation of the water pipes is neglected. |  |
| Gravity sewer pipe network: Maintenance | 3710 | meter | 1 | - | 4,90E-01 | 0,00E+00 | 0,00E+00 | 0,00E+00 | Environmental load of jetting operations is neglected. | Cost corresponds to the average O&M expenditure for the gravity sewer network of Gällivare during 2013, 2014, 2015 divided by network length (VASS, 2017). Expenditures related to pumping stations are excluded. |
| Drinking water distribution network: O&M | 3710 | meter | 1 | - | 1,33E+00 | 6,44E+01 | 4,69E-01 | 9,37E-07 | Derived from O&M costs of drinking water distribution network considering that 50% of cost is due to electrical consumption (basic process #18). | Cost corresponds to the average O&M expenditure for the drinking water distribution network of Gällivare during 2013, 2014, 2015 divided by network length (VASS, 2017). |
| District heating network: Spot repairs | 3710 | meter | 1 | - | 5,00E-01 | 5,35E-02 | 2,79E-03 | -6,28E-09 | Failure rate of 0,05 km^-1^yr^-1^ is considered for pipes from Powerpipe (Åkerström, 2004). For each failure, excavation and backfilling of 3m of trench (1,41 m^3^ of soil) is considered (basic process #1). Excavator (20 tons) is transported on site with medium weight truck over 5km (basic process #2). | Failure rate of 0,05 km-1yr-1 is considered for pipes from Powerpipe (Åkerström, 2004). Each failure leads to a repair cost of 10000€ (Yarahmadi and Sällström, 2014). |
| LTDH insulated utilidor: Production | 3710 | meter | 40 | 30 | 6,25E+01 | 3,49E+02 | 1,16E+01 | 5,50E-06 | Based on basic process #14 considering 3 kg of EPS insulation. | Price provided by Elgocell AB, supplier of low temperature district heating distribution solutions (G. Olsson, personal communication, December 14, 2016). The same lifetime as for high temperature district heating pipes is used. The amortization period of 30 years is selected in accordance with the maintenance handbook of the Swedish district heating association (SDHA, 2015). |
| LTDH insulated utilidor: Transport to construction site | 3710 | meter | 40 | 30 |  | 2,76E+00 | 1,77E-01 | 1,07E-08 | Transport from Kristianstad by heavy truck (basic process #4) of 3kg of EPS insulation. Distance is 1500km. |  |
| LTDH insulated utilidor: Installation | 3710 | meter | 40 | 30 |  | 0,00E+00 | 0,00E+00 | 0,00E+00 | The environmental load of utilidor installation is neglected. |  |
| Low temperature district heating pipes in PEX : Production | 3710 | meter | 40 | 30 |  | 1,69E+02 | 4,11E+00 | 7,37E-08 | Based on basic process #7 considering 2,1kg of PEX pipe. The production of PEX is described as the production of high density polyethylene (basic process #7). |  |
| Low temperature district heating pipes in PEX : Transport to construction site | 3710 | meter | 40 | 30 |  | 2,46E+00 | 1,58E-01 | 9,60E-09 | Transport from Kristianstad by heavy truck (basic process #4) of 2,1kg of PEX pipe. Distance is 1500km. |  |
| Low temperature district heating pipes in PEX : Installation | 3710 | meter | 40 | 30 |  | 0,00E+00 | 0,00E+00 | 0,00E+00 | The environmental load of PEX pipe installation is neglected. |  |
| Low temperature district heating pipes in PEX: Production of brass connectors | 3710 | meter | 40 | 30 |  | 1,12E+00 | 6,32E-02 | 2,24E-06 | Based on basic process #9 considering 80g of copper (Perzon et al., 2007). |  |
| Low temperature district heating pipes in PEX: transport of brass connectors to construction site | 3710 | meter | 40 | 30 |  | 1,56E-01 | 1,00E-02 | 6,08E-10 | Transport from Germany by heavy truck (basic process #4) of 80g of brass connectors. Distance is 2500km (Perzon et al., 2007). |  |
| Water-sewer insulated utilidor: Production | 3710 | meter | 40 | 30 | 2,76E+01 | 2,96E+02 | 9,80E+00 | 4,67E-06 | Based on basic process #14 considering 2,5 kg of EPS insulation. | Price provided by Elgocell AB, supplier of low temperature district heating distribution solutions (G. Olsson, personal communication, October 13, 2016). Lifetime of the low temperature district heating pipes in PEX is used. The amortization period of 30 years is selected in accordance with the maintenance handbook of the Swedish district heating association (SDHA, 2015). |
| Water-sewer insulated utilidor: Transport to construction site | 3710 | meter | 40 | 30 |  | 3,05E+00 | 1,96E-01 | 1,19E-08 | Transport from Kristianstad by heavy truck (basic process #4) of 2,5kg of EPS insulation. Distance is 1500km. |  |
| Water-sewer insulated utilidor: Installation | 3710 | meter | 40 | 30 |  | 0,00E+00 | 0,00E+00 | 0,00E+00 | The environmental load of utilidor installation is neglected. |  |
| Freeze protection pipe in PEX : Production | 3710 | meter | 40 | 30 | ↑ | 1,90E+01 | 4,61E-01 | 8,28E-09 | Based on basic process #7 considering 0,2kg of PEX pipe. The production of PEX is described as the production of high density polyethylene (basic process #7). | ↑ |
| Freeze protection pipes in PEX : Transport to construction site | 3710 | meter | 40 | 30 |  | 2,77E-01 | 1,77E-02 | 1,08E-09 | Transport from Kristianstad by heavy truck (basic process #4) of 0,2kg of PEX pipe. Distance is 1500km. |  |
| Freeze protection pipes in PEX : Installation | 3710 | meter | 40 | 30 |  | 0,00E+00 | 0,00E+00 | 0,00E+00 | The environmental load of PEX pipe installation is neglected. |  |
| Freeze protection pipe in PEX: Production of brass connectors. | 3710 | meter | 40 | 30 |  | 5,60E-01 | 3,16E-02 | 1,12E-06 | Based on basic process #9 considering 40g of copper (Perzon et al., 2007). |  |
| Freeze protection pipe in PEX: transport of brass connector to construction site | 3710 | meter | 40 | 30 |  | 7,80E-02 | 5,00E-03 | 3,04E-10 | Transport from Germany by heavy truck (basic process #4) of 40g of brass connectors. Distance is 2500km (Perzon et al., 2007). |  |
| Wastewater pumping station: Production and construction | 1 | - | 50 | 50 | 1,15E+05 | 4,28E+04 | 4,81E+03 | -2,93E-02 | 6,5m deep cylindrical excavation of diameter 2,5m for installation of the sump (basic process #1). 3,05 tonnes of stainless steel for the sump and inner wall of the pumping station; the environmental impact of conventional steel is used (basic process #10). Dimensions and mass estimates derived from (Rostfria, 2017). | Cost as provided by the water department of Gällivare municipality (Eliasson, personal communication, December 13, 2016). Lifetime of 50 years is a common value for life cycle analysis of buildings. The amortization period is collected from (Nacka, 2012) and is typical for Swedish water utilities. |
| Wastewater pumping station: Transport to construction site | 1 | - | 50 | 50 |  | 7,02E+02 | 4,50E+01 | 2,74E-06 | Based on basic process #3 considering a mass of 3 tonnes^.^ Transportation over 300km from Storfors, Sweden. |  |
| Wastewater pump: Production | 1 | - | 25 | 25 | 2,00E+04 | 9,94E+02 | 1,11E+02 | -6,16E-04 | Based on basic process #10 considering 70kg of steel and on process #9 considering 2kg of copper wire. | Price, Lifetime and amortization period as proposed by (Nacka, 2012). |
| Wastewater pump: Operation and Maintenance | 1 | - | 1 | - | 5,80E+03 | 7,03E+03 | 5,12E+01 | 1,02E-04 | Lifting of wastewater from 100 residential units over 17 meters of elevation. Consumption of electricity (basic process #18) considering a pump efficiency of 55%. Environmental load of maintenance operations is neglected. | Cost corresponds to the average O&M expenditure for sewer pumping stations of Gällivare during 2013, 2014, 2015 divided by the number of pumping station in Gällivare (VASS, 2017). |
| District heating substation single family home: Production and installation | 71 | - | 20 | 20 | 1,50E+03 | 8,80E+02 | 6,08E+01 | 2,16E-02 | Based on process 8.1.01 "Substation district heating (A1-A3), 1 kW" from Oekobaudat 2016 database (BMUB, 2016) considering a capacity of 10kW. | Cost as estimated by the Danish Energy Agency (Energistyrelsen, 2012). Lifetime of 20 years is suggested by both the Oekobaudat 2016 database (BMUB, 2016) and the Danish energy agency (Energistyrelsen, 2012). Amortization period of 20 years is suggested by the Swedish district heating association (SDHA, 2007). |
| District heating substation single family home: Transport to construction site | 71 | - | 20 | 20 |  | 1,37E+01 | 8,75E-01 | 5,32E-08 | Substations are transported from Helsinge, Denmark (Perzon et al., 2007) by heavy weight truck (basic process #3). Distance is 1750 km and weight is 10kg (1kg/kW as provided by the Oekobaudat 2016 database (BMUB, 2016)). |  |
| District heating substation single family home: Maintenance | 71 | - | 1 | - | 1,50E+02 | 0,00E+00 | 0,00E+00 | 0,00E+00 | Environmental load of district heating substations maintenance is neglected. | Cost as estimated by the Danish Energy Agency (Energistyrelsen, 2012). |
| District heating substation 20 apartments building: Production | 7 | - | 20 | 20 | 7,00E+03 | 8,80E+03 | 6,08E+02 | 2,16E-01 | Based on process 8.1.01 "Substation district heating (A1-A3), 1 kW" from Oekobaudat 2016 database (BMUB, 2016) considering a capacity of 100kW. | An investment cost of 70€/kW (Energistyrelsen, 2012) was used considering a capacity of 100kW. Lifetime of 20 years is suggested by both the Oekobaudat 2016 database (BMUB, 2016) and the Danish energy agency (Energistyrelsen, 2012). Amortization period of 20 years is suggested by the Swedish district heating association (SDHA, 2007). |
| District heating substation 20 apartments building: Transport to construction site | 7 | - | 20 | 20 |  | 1,37E+02 | 8,75E+00 | 5,32E-07 | Substations are transported from Helsinge, Denmark (Perzon et al., 2007) by heavy weight truck (basic process #3). Distance is 1750 km and weight is 100kg (1kg/kW as provided by the Oekobaudat database (BMUB, 2016)). |  |
| District heating substation 20 apartments building: Maintenance | 7 | - | 1 | - | 5,00E+02 | 0,00E+00 | 0,00E+00 | 0,00E+00 | Environmental load of district heating substations maintenance is neglected. | Cost as estimated by the Danish Energy Agency (Energistyrelsen, 2012). |
| Low temperature district heating house station : Production of elements and construction | 1 | - | 50 | 30 | 2,00E+04 | 0,00E+00 | 0,00E+00 | 0,00E+00 | Environmental load of production and constriction of the district heating house station is neglected. | Price estimate provided by Elgocell AB (Göran Olsson, personal communication, August 2017). Lifetime of 50 years is a common value for life cycle analysis of buildings. The amortization period suggested by (Svensk Fjärrvärme, 2015) for district heating networks is used. |
| Heat exchanger HT-LT and pumps: Manufacturing | 1 | - | 20 | 20 | 2,00E+04  ↑ | 1,24E+05 | 8,57E+03 | 3,05E+00 | Based on process 8.1.01 "Substation district heating (A1-A3), 1 kW" from Oekobaudat 2016 database (BMUB, 2016) considering a capacity of 1410kW. | Price estimate provided by Elgocell AB (Göran Olsson, personal communication, August 2017). Lifetime and amortization period of a district heating substations are used.  ↑ |
| Heat exchanger HT-LT and pumps: Transport | 1 | - | 20 | 20 |  | 2,75E+03 | 1,76E+02 | 1,07E-05 | Substations are transported from Germany (Perzon et al., 2007) by heavy weight truck (basic process #3). Distance is 2500 km and weight is 1410kg (1kg/kW as provided by the Oekobaudat database (BMUB, 2016)). |  |
| Low temperature district heating network: Production of heat lost to surrounding soil | 3710 | meter | 1 | - | 1,21E+00 | 2,77E+02 | 6,48E+00 | 1,91E-06 | Heat loss rate of 6 W/m (average over the year) as provided by Elgocell AB (G. Olsson, personal communication, October 13, 2016) for a similar project in Kiruna, Sweden. District heat from Gällivare CHP plant is consumed: 46% peat (basic process #20) and 54% wood chips (basic process #21). Fuel mix is derived from the annual report 2015 of Gällivare energy company (GEAB, 2016). | Heat loss rate of 6 W/m (average over the year) as provided by Elgocell AB (G. Olsson, personal communication, October 13, 2016) for a similar project in Kiruna, Sweden. District heat from Gällivare CHP plant is consumed at a marginal cost of 0,02 €/kWh. Marginal cost is derived from the annual report 2015 of Gällivare energy company (GEAB, 2016) by dividing annual O&M costs for heat production (including fuel costs) by annual heat production. |
| Water-sewer insulated utilidor: freeze protection | 3710 | meter | 1 | - | 3,90E-01 | 8,96E+01 | 2,10E+00 | 6,19E-07 | Use of 6W/m of district heat during 6 months for freeze protection of water and sewer pipe. The heat tracing power of 6 W/m is estimated with the method proposed by (Gunderson, 1978). District heat from Gällivare CHP plant is consumed: 46% peat (basic process #20) and 54% wood chips (basic process #21). Fuel mix is derived from the annual report 2015 of Gällivare energy company (GEAB, 2016). | Use of 6W/m of district heat during 6 months for freeze protection of water and sewer pipe. The heat tracing power of 6 W/m is estimated with the method proposed by (Gunderson, 1978). District heat from Gällivare CHP plant is consumed at a marginal cost of 0,02 €/kWh. Marginal cost is derived from the annual report 2015 of Gällivare energy company (GEAB, 2016) by dividing annual O&M costs for heat production (including fuel costs) by annual heat production. |
| Heat provided to single family home: Production at district heating plant | 71 | - | 1 | - | 3,81E+02 | 8,76E+04 | 2,05E+03 | 6,05E-04 | Annual heat demand of 16900 kWh is considered. District heat from Gällivare CHP plant is consumed: 46% peat (basic process #20) and 54% wood chips (basic process #21). Fuel mix is derived from the annual report 2015 of Gällivare energy company (GEAB, 2016). | Annual heat demand of 16900 kWh is considered. District heat from Gällivare CHP plant is consumed at a marginal cost of 0,02 €/kWh. Marginal cost is derived from the annual report 2015 of Gällivare energy company (GEAB, 2016) by dividing annual O&M costs for heat production (including fuel costs) by annual heat production. |
| Heat delivered to 20 apartments building: Production at district heating plant | 7 | - | 1 | - | 3,63E+03 | 8,35E+05 | 1,95E+04 | 5,77E-03 | Annual heat demand of 161000 kWh is considered (20*8050kWh). District heat from Gällivare CHP plant is consumed: 46% peat (basic process #20) and 54% wood chips (basic process #21). Fuel mix is derived from the annual report 2015 of Gällivare energy company (GEAB, 2016). | Annual heat demand of 161000 kWh is considered (20*8050kWh). District heat from Gällivare CHP plant is consumed at a marginal cost of 0,02 €/kWh. Marginal cost is derived from the annual report 2015 of Gällivare energy company (GEAB, 2016) by dividing annual O&M costs for heat production (including fuel costs) by annual heat production. |

Table S4. Output flow, flow unit, lifetime, amortization period, cost and environmental impacts of the system processes composing alternative 3 (low pressure sewer and low temperature district heating).

| **System process** | **Output flow** | **Flow unit** | **Lifetime** | **Amortizati-on period** | **Cost** | **Cumulative Exergy Demand CExD** | **Global Warming Potential GWP** | **Abiotic Depletion Potential of Elements ADPE** | **Data sources and assumptions** | |
| --- | --- | --- | --- | --- | --- | --- | --- | --- | --- | --- |
|  |  |  | *Years* | *Years* | €*/unit* | *MJ/unit* | *KgCO_2_Eq/unit* | *KgSbEq/unit* | **Unitary environmental impacts (CExD, GWP, ADPE)** | **Unitary cost, lifetime and amortization period** |
| Trench for LTDH and LPS: Excavation with a 100kW excavator. | 3510 | meter | 40 | 30 | 7,00E+01 | 1,45E+01 | 9,74E-01 | 4,99E-08 | Based on basic process #1 considering 0,75 m^3^ of soil. | Cost of a shallow trench including installation of water and sewer pipes as provided by the water utility of Kiruna (Skoglind, 2014). Kiruna is a municipality nearby Gällivare with similar labour costs. Lifetime of low temperature district heating pipes in PEX in used. The amortization period of 30 years is selected in accordance with the maintenance handbook of the Swedish district heating association (SDHA, 2015). |
| Trench for LTDH and LPS: Backfilling with a 100kW excavator. | 3510 | meter | 40 | 30 |  | 9,66E+00 | 6,49E-01 | 3,33E-08 | Based on basic process #1 considering 0,5 m^3^ of soil. |  |
| Crushed rocks 0-8mm: Production | 3510 | meter | 40 | 30 |  | 2,90E+01 | 7,58E-01 | 1,52E-06 | Based on basic process #6 considering 0,48 m^3^ of 0-8mm crushed rocks. |  |
| Crushed rocks 0-8mm: Transport to construction site | 3510 | meter | 40 | 30 |  | 1,10E+01 | 7,09E-01 | 4,32E-08 | Based on basic process #2 considering 0,48 m^3^ of 0-8mm crushed rocks at density 1400 kg/m^3.^ Transportation over 16km from LKAB Malmberget mine. |  |
| Remaining natural soil: Transport to disposal site | 3510 | meter | 40 | 30 |  | 5,96E+00 | 3,85E-01 | 2,35E-08 | Based on basic process #2 considering 0,73 m^3^ of natural soil at density 1600 kg/m^3.^ Transportation over 5km to disposal site. |  |
| LPS pipe in PE: Production | 3510 | meter | 40 | 30 |  | 8,07E+01 | 1,96E+00 | 3,52E-08 | Based on basic process #7 considering 1kg of PE. Only the production of raw material is considered as it represents most of the environmental load of PE pipes manufacturing (Carolin et al., 2011). |  |
| LPS pipe in PE: Transport to construction site | 3510 | meter | 40 | 30 |  | 2,67E-01 | 1,72E-02 | 1,05E-09 | Based on basic process #2 considering 1kg of PE pipe. Transportation over 260km from Pipelife AB facilities in Haparanda, Sweden. |  |
| LPS pipe in PE: Installation | 3510 | meter | 125 | 50 |  | 0,00E+00 | 0,00E+00 | 0,00E+00 | Environmental load of the installation of the LPS pipes is neglected. |  |
| Drinking water pipe in PE: Production | 3510 | meter | 125 | 50 |  | 1,59E+02 | 3,88E+00 | 6,96E-08 | Based on basic process #7 considering 1,98kg of PE. Only the production of raw material is considered as it represents most of the environmental load of PE pipes manufacturing (Carolin et al., 2011). |  |
| Drinking water pipe in PE: Transport to construction site | 3510 | meter | 125 | 50 |  | 5,27E-01 | 3,41E-02 | 2,08E-09 | Based on basic process #2 considering 1,98kg of PE pipe. Transportation over 260km from Pipelife AB facilities in Haparanda, Sweden. |  |
| Drinking water pipe in PE: Installation | 3510 | meter | 125 | 50 |  | 0,00E+00 | 0,00E+00 | 0,00E+00 | Environmental load of the installation of the water pipes is neglected. |  |
| Drinking water distribution network: O&M | 3510 | meter | 1 | - | 1,33E+00 | 6,44E+01 | 4,69E-01 | 9,37E-07 | Derived from O&M costs of drinking water distribution network considering that 50% of cost is due to electrical consumption (basic process #18). | Cost corresponds to the average O&M expenditure for the drinking water distribution network of Gällivare during 2013, 2014, 2015 divided by network length (VASS, 2017). |
| District heating network: Spot repairs | 3510 | meter | 1 | - | 5,00E-01 | 5,35E-02 | 2,79E-03 | -6,28E-09 | Failure rate of 0,05 km^-1^yr^-1^ is considered for pipes from Powerpipe (Åkerström, 2004). For each failure, excavation and backfilling of 3m of trench (1,41 m^3^ of soil) is considered (basic process #1). Excavator (20 tons) is transported on site with medium weight truck over 5km (basic process #2). | Failure rate of 0,05 km-1yr-1 is considered for pipes from Powerpipe (Åkerström, 2004). Each failure leads to a repair cost of 10000€ (Yarahmadi and Sällström, 2014). |
| LTDH insulated utilidor: Production | 3510 | meter | 40 | 30 | 6,25E+01  ↑ | 3,49E+02 | 1,16E+01 | 5,50E-06 | Based on basic process #14 considering 3 kg of EPS insulation. | Price provided by Elgocell AB, supplier of low temperature district heating distribution solutions (G. Olsson, personal communication, December 14, 2016). The same lifetime as for high temperature district heating pipes is used. The amortization period of 30 years is selected in accordance with the maintenance handbook of the Swedish district heating association (SDHA, 2015).  ↑ |
| LTDH insulated utilidor: Transport to construction site | 3510 | meter | 40 | 30 |  | 2,76E+00 | 1,77E-01 | 1,07E-08 | Transport from Kristianstad by heavy truck (basic process #4) of 3kg of EPS insulation. Distance is 1500km. |  |
| LTDH insulated utilidor: Installation | 3510 | meter | 40 | 30 |  | 0,00E+00 | 0,00E+00 | 0,00E+00 | The environmental load of utilidor installation is neglected. |  |
| Low temperature district heating pipes in PEX : Production | 3510 | meter | 40 | 30 |  | 1,69E+02 | 4,11E+00 | 7,37E-08 | Based on basic process #7 considering 2,1kg of PEX pipe. The production of PEX is described as the production of high density polyethylene (basic process #7). |  |
| Low temperature district heating pipes in PEX : Transport to construction site | 3510 | meter | 40 | 30 |  | 2,46E+00 | 1,58E-01 | 9,60E-09 | Transport from Kristianstad by heavy truck (basic process #4) of 2,1kg of PEX pipe. Distance is 1500km. |  |
| Low temperature district heating pipes in PEX : Installation | 3510 | meter | 40 | 30 |  | 0,00E+00 | 0,00E+00 | 0,00E+00 | The environmental load of PEX pipe installation is neglected. |  |
| Low temperature district heating pipes in PEX: Production of brass connectors | 3510 | meter | 40 | 30 |  | 1,12E+00 | 6,32E-02 | 2,24E-06 | Based on basic process #9 considering 80g of copper (Perzon et al., 2007). |  |
| Low temperature district heating pipes in PEX: transport of brass connectors to construction site | 3510 | meter | 40 | 30 |  | 1,56E-01 | 1,00E-02 | 6,08E-10 | Transport from Germany by heavy truck (basic process #4) of 80g of brass connectors. Distance is 2500km (Perzon et al., 2007). |  |
| Water-sewer insulated utilidor: Production | 3510 | meter | 40 | 30 | 2,76E+01 | 2,96E+02 | 9,80E+00 | 4,67E-06 | Based on basic process #14 considering 2,5 kg of EPS insulation. | Price provided by Elgocell AB, supplier of low temperature district heating distribution solutions (G. Olsson, personal communication, October 13, 2016). Lifetime of the low temperature district heating pipes in PEX is used. The amortization period of 30 years is selected in accordance with the maintenance handbook of the Swedish district heating association (SDHA, 2015). |
| Water-sewer insulated utilidor: Transport to construction site | 3510 | meter | 40 | 30 |  | 3,05E+00 | 1,96E-01 | 1,19E-08 | Transport from Kristianstad by heavy truck (basic process #4) of 2,5kg of EPS insulation. Distance is 1500km. |  |
| Water-sewer insulated utilidor: Installation | 3510 | meter | 40 | 30 |  | 0,00E+00 | 0,00E+00 | 0,00E+00 | The environmental load of utilidor installation is neglected. |  |
| Freeze protection pipe in PEX : Production | 3510 | meter | 40 | 30 |  | 1,90E+01 | 4,61E-01 | 8,28E-09 | Based on basic process #7 considering 0,2kg of PEX pipe. The production of PEX is described as the production of high density polyethylene (basic process #7). |  |
| Freeze protection pipes in PEX : Transport to construction site | 3510 | meter | 40 | 30 |  | 2,77E-01 | 1,77E-02 | 1,08E-09 | Transport from Kristianstad by heavy truck (basic process #4) of 0,2kg of PEX pipe. Distance is 1500km. |  |
| Freeze protection pipes in PEX : Installation | 3510 | meter | 40 | 30 |  | 0,00E+00 | 0,00E+00 | 0,00E+00 | The environmental load of PEX pipe installation is neglected. |  |
| Freeze protection pipe in PEX: Production of brass connectors. | 3510 | meter | 40 | 30 |  | 5,60E-01 | 3,16E-02 | 1,12E-06 | Based on basic process #9 considering 40g of copper (Perzon et al., 2007). |  |
| Freeze protection pipe in PEX: transport of brass connector to construction site | 3510 | meter | 40 | 30 |  | 7,80E-02 | 5,00E-03 | 3,04E-10 | Transport from Germany by heavy truck (basic process #4) of 40g of brass connectors. Distance is 2500km (Perzon et al., 2007). |  |
| LPS tank for single family home : Production | 71 | - | 125 | 25 | 6,00E+02 | 5,61E+03 | 1,37E+02 | 2,45E-06 | Based on basic process #7 considering 70kg of PE. Only the production of raw material is considered. Weight of tank obtained directly from “Skandinavisk Kommunalteknik AB”, supplier of LPS solutions (T. Jansson, personal communication, November 17, 2016). | Costs as provided from “Skandinavisk Kommunalteknik AB”, supplier of LPS solutions (T. Jansson, personal communication, November 17, 2016). The same lifetime as for PE pipes in Sweden is used since the tanks are also made of PE and installed in the ground. Amortization period is obtained from (Nacka, 2012). |
| LPS tank for single family home : Transport to construction site | 71 | - | 125 | 25 |  | 5,84E+01 | 3,75E+00 | 2,28E-07 | Based on basic process #3 considering 70kg of PE. Transport over 1070km from Uppsala, Sweden. |  |
| LPS tank for single family home : Installation | 71 | - | 125 | 25 | 7,46E+02 | 1,45E+03 | 9,73E+01 | 4,99E-06 | Based on basic process #1 considering an excavation of 37,7m^3^ of sandy soil and backfilling of 36,9m^3^. Remaining soil (0,8m^3^) is transported by medium weight truck over 5 km to a disposal site (basic process #2). | Excavation of 37,7m^3^ of soil at 10€/m^3^ and backfilling of 36,9m^3^ of soil at 10€/m^3^. Excavation cost is provided by Gällivare municipality (Eliasson, personal communication, December 13, 2016). Lifetime and amortization period of the LPS tank are used. |
| LPS tanks for 20 apartments building : Production | 7 | - | 125 | 25 | 4,80E+03 | 1,99E+04 | 9,58E+02 | 7,29E-04 | 3 tanks of 180kg made of glass reinforced polyester (GRP). Production of raw material is modelled thought basic processes #15 #16 and #17 considering 500g of glass fibres, 320g of polyester resin and 160g of calcium carbonate per kg of GRP (Wang et al., 2013). | 1600€ per tank as provided by “Skandinavisk Kommunalteknik AB”, supplier of LPS solutions (T. Jansson, personal communication, November 17, 2016). Lifetime and amortization lifetime: same as LPS tank for single family home. |
| LPS tanks for 20 apartments building : Transport to construction site | 7 | - | 125 | 25 |  | 4,51E+02 | 2,89E+01 | 1,76E-06 | Based on basic process #3 considering 3*180kg of glass reinforced polyester. Transport over 1070km from Uppsala, Sweden. |  |
| LPS tanks for 20 apartments building : Installation | 7 | - | 125 | 25 | 2,77E+03 | 5,43E+03 | 3,65E+02 | 1,88E-05 | Based on basic process #1 considering an excavation of 143m^3^ of sandy soil and backfilling of 134m^3^. Remaining soil (9m^3^) is transported by medium weight truck over 5 km to a disposal site (basic process #2). | Excavation of 143m^3^ of soil at 10€/m^3^ and backfilling of 134m^3^ of soil at 10€/m^3^. . Excavation cost is provided by Gällivare municipality (Eliasson, personal communication, December 13, 2016). Lifetime and amortization period of the LPS tank are used. |
| LPS pump for single family home : Production | 71 | - | 25 | 25 | 1,70E+03  ↑ | 6,63E+02 | 7,26E+01 | -3,48E-04 | Based on basic process #10 and #9 considering 45kg of cast iron (environmental load of steel is used) and 3kg of copper wire. Mass estimates obtained from the supplier “Skandinavisk Kommunalteknik AB” (T. Jansson, personal communication, November 17, 2016). | Price as provided by “Skandinavisk Kommunalteknik AB”, supplier of LPS solutions (T. Jansson, personal communication, November 17, 2016). Lifetime and amortization period as suggested by Nacka municipality (Nacka, 2012).  ↑ |
| LPS pump for single family home : Transport to construction site | 71 | - | 25 | 25 |  | 1,08E+02 | 6,48E+00 | 4,22E-07 | Transport of the 48kg pump over 6000km by sea cargo (basic process #3) from New York to Gothenburg. Transport over 1500 km by heavy truck (basic process #4) from Gothenburg to Gällivare. |  |
| LPS pump for single family home : Installation | 71 | - | 25 | 25 |  | 0,00E+00 | 0,00E+00 | 0,00E+00 | The environmental load of pump installation is neglected. |  |
| LPS pump for single family home : Operation | 71 | - | 1 | - | 3,91E+00 | 3,78E+02 | 2,75E+00 | 5,50E-06 | Use of 50 kWh per year of electricity (basic process #18) as provided by (Lindqvist et al., 2000). | Use of 50 kWh per year of electricity as provided by (Lindqvist et al., 2000). Electricity price of 0,078 €/kWh (source:Vattenfall) is used. |
| LPS pump for single family home : Maintenance | 71 | - | 1 | - | 1,50E+01 | 0,00E+00 | 0,00E+00 | 0,00E+00 | The environmental load of pump maintenance is neglected. | Maintenance operation needs to be done in average 1,5 times during the 25 years life cycle of the pump and costs 250€. Data communicated by the supplier “Skandinavisk Kommunalteknik AB” (T. Jansson, personal communication, November 17, 2016). |
| LPS pumps for 20 apartments building : Production | 7 | - | 25 | 25 | 1,70E+04 | 6,63E+03 | 7,26E+02 | -3,48E-03 | Based on basic process #10 and #9 considering 10*45kg of cast iron (environmental load of steel is used) and 10*3kg of copper wire. Mass estimates obtained from the supplier “Skandinavisk Kommunalteknik AB” (T. Jansson, personal communication, November 17, 2016). | 10 pumps with unitary cost 1700€ as provided by “Skandinavisk Kommunalteknik AB”, supplier of LPS solutions (T. Jansson, personal communication, November 17, 2016). Lifetime and amortization period as suggested by Nacka municipality (Nacka, 2012). |
| LPS pumps for 20 apartments building : Transport to construction site | 7 | - | 25 | 25 |  | 1,08E+03 | 6,48E+01 | 4,22E-06 | Transport of 10 pumps (10*48kg) over 6000km by sea cargo (basic process #3) from New York to Gothenburg. Transport over 1500 km by heavy truck (basic process #4) from Gothenburg to Gällivare. |  |
| LPS pumps for 20 apartments building : Installation | 7 | - | 25 | 25 |  | 0,00E+00 | 0,00E+00 | 0,00E+00 | The environmental load of pump installation is neglected. |  |
| LPS pumps for 20 apartments building : Operation | 7 | - | 1 | - | 7,81E+01 | 7,56E+03 | 5,50E+01 | 1,10E-04 | Use of 20*50 kWh per year of electricity (basic process #18). | Use of 20*50 kWh per year of electricity at a price of 0,078 €/kWh (source:Vattenfall). |
| LPS pumps for 20 apartments building : Maintenance | 7 | - | 1 | - | 1,50E+02 | 0,00E+00 | 0,00E+00 | 0,00E+00 | The environmental load of pump maintenance is neglected. | Maintenance operation needs to be done in average 1,5 times during the 25 years life cycle of each pump and costs 250€. 10 pumps are used for a 20 apartment building. Data communicated by “Skandinavisk Kommunalteknik AB” (T. Jansson, personal communication, November 17, 2016). |
| District heating substation single family home: Production and installation | 71 | - | 20 | 20 | 1,50E+03 | 8,80E+02 | 6,08E+01 | 2,16E-02 | Based on process 8.1.01 "Substation district heating (A1-A3), 1 kW" from Oekobaudat 2016 database (BMUB, 2016) considering a capacity of 10kW. | Cost as estimated by the Danish Energy Agency (Energistyrelsen, 2012). Lifetime of 20 years is suggested by both the Oekobaudat 2016 database (BMUB, 2016) and the Danish energy agency (Energistyrelsen, 2012). Amortization period of 20 years is suggested by the Swedish district heating association (SDHA, 2007). |
| District heating substation single family home: Transport to construction site | 71 | - | 20 | 20 |  | 1,37E+01 | 8,75E-01 | 5,32E-08 | Substations are transported from Helsinge, Denmark (Perzon et al., 2007) by heavy weight truck (basic process #3). Distance is 1750 km and weight is 10kg (1kg/kW as provided by the Oekobaudat 2016 database (BMUB, 2016)). |  |
| District heating substation single family home: Maintenance | 71 | - | 1 | - | 1,50E+02 | 0,00E+00 | 0,00E+00 | 0,00E+00 | Environmental load of district heating substations maintenance is neglected. | Cost as estimated by the Danish Energy Agency (Energistyrelsen, 2012). |
| District heating substation 20 apartments building: Production | 7 | - | 20 | 20 | 7,00E+03 | 8,80E+03 | 6,08E+02 | 2,16E-01 | Based on process 8.1.01 "Substation district heating (A1-A3), 1 kW" from Oekobaudat 2016 database (BMUB, 2016) considering a capacity of 100kW. | An investment cost of 70€/kW (Energistyrelsen, 2012) was used considering a capacity of 100kW. Lifetime of 20 years is suggested by both the Oekobaudat 2016 database (BMUB, 2016) and the Danish energy agency (Energistyrelsen, 2012). Amortization period of 20 years is suggested by the Swedish district heating association (SDHA, 2007). |
| District heating substation 20 apartments building: Transport to construction site | 7 | - | 20 | 20 |  | 1,37E+02 | 8,75E+00 | 5,32E-07 | Substations are transported from Helsinge, Denmark (Perzon et al., 2007) by heavy weight truck (basic process #3). Distance is 1750 km and weight is 100kg (1kg/kW as provided by the Oekobaudat database). |  |
| District heating substation 20 apartments building: Maintenance | 7 | - | 1 | - | 5,00E+02 | 0,00E+00 | 0,00E+00 | 0,00E+00 | Environmental load of district heating substations maintenance is neglected. | Cost as estimated by the Danish Energy Agency (Energistyrelsen, 2012). |
| Low temperature district heating house station : Production of elements and construction | 1 | - | 50 | 30 | 2,00E+04 | 0,00E+00 | 0,00E+00 | 0,00E+00 | Environmental load of production and constriction of the district heating house station is neglected. | Price estimate provided by Elgocell AB (Göran Olsson, personal communication, August 2017). Lifetime of 50 years is a common value for life cycle analysis of buildings. The amortization period suggested by (Svensk Fjärrvärme, 2015) for district heating networks is used. |
| Heat exchanger HT-LT and pumps: Manufacturing | 1 | - | 40 | 30 | 2,00E+04 | 1,24E+05 | 8,57E+03 | 3,05E+00 | Based on process 8.1.01 "Substation district heating (A1-A3), 1 kW" from Oekobaudat 2016 database (BMUB, 2016) considering a capacity of 1410kW. | Price estimate provided by Elgocell AB (Göran Olsson, personal communication, August 2017). Lifetime and amortization period of a district heating substations are used. |
| Heat exchanger HT-LT and pumps: Transport | 1 | - | 40 | 30 |  | 2,75E+03 | 1,76E+02 | 1,07E-05 | Substations are transported from Germany (Perzon et al., 2007) by heavy weight truck (basic process #3). Distance is 2500 km and weight is 1410kg (1kg/kW as provided by the Oekobaudat database (BMUB, 2016)). |  |
| Low temperature district heating network: production of heat lost to surrounding soil | 3510 | meter | 1 | - | 1,21E+00 | 2,77E+02 | 6,48E+00 | 1,91E-06 | Heat loss rate of 6 W/m (average over the year) as provided by Elgocell AB (G. Olsson, personal communication, October 13, 2016) for a similar project in Kiruna, Sweden. District heat from Gällivare CHP plant is consumed: 46% peat (basic process #20) and 54% wood chips (basic process #21). Fuel mix is derived from the annual report 2015 of Gällivare energy company (GEAB, 2016). | Heat loss rate of 6 W/m (average over the year) as provided by Elgocell AB (G. Olsson, personal communication, October 13, 2016) for a similar project in Kiruna, Sweden. District heat from Gällivare CHP plant is consumed at a marginal cost of 0,02 €/kWh. Marginal cost is derived from the annual report 2015 of Gällivare energy company (GEAB, 2016) by dividing annual O&M costs for heat production (including fuel costs) by annual heat production. |
| Water-sewer insulated utilidor: Freeze protection | 3510 | meter | 1 | - | 3,90E-01 | 8,96E+01 | 2,10E+00 | 6,19E-07 | Use of 6W/m of district heat during 6 months for freeze protection of water and sewer pipe. The heat tracing power of 6 W/m is estimated with the method proposed by (Gunderson, 1978). District heat from Gällivare CHP plant is consumed: 46% peat (basic process #20) and 54% wood chips (basic process #21). Fuel mix is derived from the annual report 2015 of Gällivare energy company (GEAB, 2016). | Use of 6W/m of district heat during 6 months for freeze protection of water and sewer pipe. The heat tracing power of 6 W/m is estimated with the method proposed by (Gunderson, 1978). District heat from Gällivare CHP plant is consumed at a marginal cost of 0,02 €/kWh. Marginal cost is derived from the annual report 2015 of Gällivare energy company (GEAB, 2016) by dividing annual O&M costs for heat production (including fuel costs) by annual heat production. |
| Heat provided to single family home: Production at district heating plant | 71 | - | 1 | - | 3,81E+02 | 8,76E+04 | 2,05E+03 | 6,05E-04 | Annual heat demand of 16900 kWh is considered. District heat from Gällivare CHP plant is consumed: 46% peat (basic process #20) and 54% wood chips (basic process #21). Fuel mix is derived from the annual report 2015 of Gällivare energy company (GEAB, 2016). | Annual heat demand of 16900 kWh is considered. District heat from Gällivare CHP plant is consumed at a marginal cost of 0,02 €/kWh. Marginal cost is derived from the annual report 2015 of Gällivare energy company (GEAB, 2016) by dividing annual O&M costs for heat production (including fuel costs) by annual heat production. |
| Heat delivered to 20 apartments building: Production at district heating plant | 7 | - | 1 | - | 3,63E+03 | 8,35E+05 | 1,95E+04 | 5,77E-03 | Annual heat demand of 161000 kWh is considered (20*8050kWh). District heat from Gällivare CHP plant is consumed: 46% peat (basic process #20) and 54% wood chips (basic process #21). Fuel mix is derived from the annual report 2015 of Gällivare energy company (GEAB, 2016). | Annual heat demand of 161000 kWh is considered (20*8050kWh). District heat from Gällivare CHP plant is consumed at a marginal cost of 0,02 €/kWh. Marginal cost is derived from the annual report 2015 of Gällivare energy company (GEAB, 2016) by dividing annual O&M costs for heat production (including fuel costs) by annual heat production. |

Table S5. Output flow, flow unit, lifetime, amortization period, cost and environmental impacts of the system processes composing alternative 4 (gravity sewer and geothermal heat pumps).

| **System process** | **Output flow** | **Flow unit** | **Lifetime** | **Amortizati-on period** | **Cost** | **Cumulative Exergy Demand CExD** | **Global Warming Potential GWP** | **Abiotic Depletion Potential of Elements ADPE** | **Data sources and assumptions** | |
| --- | --- | --- | --- | --- | --- | --- | --- | --- | --- | --- |
|  |  |  | *Years* | *Years* | €*/unit* | *MJ/unit* | *KgCO_2_Eq/unit* | *KgSbEq/unit* | **Unitary environmental impacts (CExD, GWP, ADPE)** | **Unitary cost, lifetime and amortization period** |
| Trench to frost-free depth for sanitary pipes: Excavation with a 100kW excavator. | 3710 | meter | 125 | 50 | 4,70E+02 | 7,69E+01 | 5,17E+00 | 2,65E-07 | Based on basic process #1 considering 3,98 m^3^ of soil. | Cost as provided by “WSP Samhällsbyggnad Norrbotten”, consulting company regularly contracted by the water department of Gällivare municipality (L. Björnfot, personal communication, December 09, 2016). Lifetime is the average survival time for new sewer pipes according to the survival function proposed by (Malm et al., 2013).The amortization period is collected from (Nacka, 2012) and is typical for Swedish water utilities. |
| Trench to frost-free depth for sanitary pipes: Backfilling with a 100kW excavator. | 3710 | meter | 125 | 50 |  | 7,63E+01 | 5,13E+00 | 2,63E-07 | Based on basic process #1 considering 3,95 m^3^ of soil. |  |
| Crushed rock 0-8mm: Production | 3710 | meter | 125 | 50 |  | 7,78E+01 | 2,04E+00 | 4,08E-06 | Based on basic process #6 considering 1,29 m^3^ of 0-8mm crushed rocks. |  |
| Crushed rock 0-8mm: Transport to construction site | 3710 | meter | 125 | 50 |  | 2,95E+01 | 1,91E+00 | 1,16E-07 | Based on basic process #2 considering 1,29 m^3^ of 0-8mm crushed rocks at density 1400 kg/m^3.^ Transportation over 16km from LKAB Malmberget mine. |  |
| Crushed rock 16-32mm: Production | 3710 | meter | 125 | 50 |  | 6,73E+01 | 1,76E+00 | 3,53E-06 | Based on basic process #5 considering 1,59 m^3^ of 16-32mm crushed rocks. |  |
| Crushed rock 16-32mm: Transport to construction site | 3710 | meter | 125 | 50 |  | 3,63E+01 | 2,35E+00 | 2,66E-07 | Based on basic process #2 considering 1,59 m^3^ of 16-32mm crushed rocks at density 1400 kg/m^3.^ Transportation over 16km from LKAB Malmberget mine. |  |
| Remaining natural soil: Transport to disposal site | 3710 | meter | 125 | 50 |  | 2,37E+01 | 1,54E+00 | 9,36E-08 | Based on basic process #2 considering 2,91 m^3^ of natural soil at density 1600 kg/m^3.^ Transportation over 5km to disposal site. |  |
| Gravity sewer pipe in PVC: Production | 3710 | meter | 125 | 50 |  | 2,35E+02 | 1,03E+01 | 6,45E-05 | Based on basic process #11 considering 3,8kg of PVC. Only the production of raw material is considered as it represents most of the environmental load of PVC sewer pipes manufacturing (Carolin et al., 2012). |  |
| Gravity sewer pipe in PVC: Transport to construction site | 3710 | meter | 125 | 50 |  | 1,01E+00 | 6,51E-02 | 3,97E-09 | Based on basic process #2 considering 3,8kg of PVC pipe. Transportation over 260km from Pipelife AB facilities in Haparanda, Sweden. |  |
| Manhole in PP: production | 3710 | meter | 125 | 50 |  | 8,89E+01 | 2,27E+00 | 6,10E-08 | Based on basic process #8 assuming one manhole every 45m of sewer pipe corresponding to 1,15kg of PP per meter of pipe (Carolin et al., 2012). Only the production of raw material is considered. |  |
| Manhole in PP: transport to construction site | 3710 | meter | 125 | 50 | ↑ | 3,05E-01 | 1,97E-02 | 1,20E-09 | Based on basic process #2 considering 1,15kg of PP manhole. Transportation over 260km from Pipelife AB facilities in Haparanda, Sweden. | ↑ |
| Pressurized sewer pipe in PE: Production | 3710 | meter | 125 | 50 |  | 1,32E+02 | 3,22E+00 | 5,77E-08 | Based on basic process #7 considering 1,65kg of PE. Only the production of raw material is considered as it represents most of the environmental load of PE pipes manufacturing (Carolin et al., 2011). |  |
| Pressurized sewer pipe in PE: Transport | 3710 | meter | 125 | 50 |  | 4,37E-01 | 2,83E-02 | 1,72E-09 | Based on basic process #2 considering 1,65kg of PE sewer pipe. Transportation over 260km from Pipelife AB facilities in Haparanda, Sweden. |  |
| Drinking water pipe in PE: Production | 3710 | meter | 125 | 50 |  | 1,59E+02 | 3,88E+00 | 6,96E-08 | Based on basic process #7 considering 1,98kg of PE. Only the production of raw material is considered as it represents most of the environmental load of PE pipes manufacturing (Carolin et al., 2011). |  |
| Drinking water pipe in PE: Transport to construction site | 3710 | meter | 125 | 50 |  | 5,27E-01 | 3,41E-02 | 2,08E-09 | Based on basic process #2 considering 1,98kg of PE pipe. Transportation over 260km from Pipelife AB facilities in Haparanda, Sweden. |  |
| Drinking water pipe in PE: Installation | 3710 | meter | 125 | 50 |  | 0,00E+00 | 0,00E+00 | 0,00E+00 | Environmental load of the installation of the water pipes is neglected. |  |
| Gravity sewer pipe network: Maintenance | 3710 | meter | 1 | - | 4,90E-01 | 0,00E+00 | 0,00E+00 | 0,00E+00 | Environmental load of jetting operations is neglected. | Cost corresponds to the average O&M expenditure for the gravity sewer network of Gällivare during 2013, 2014, 2015 divided by network length (VASS, 2017). Expenditures related to pumping stations are excluded. |
| Wastewater pumping station: Production and construction | 1 | - | 50 | 50 | 1,15E+05 | 4,28E+04 | 4,81E+03 | -2,93E-02 | 6,5m deep cylindrical excavation of diameter 2,5m for installation of the sump (basic process #1). 3,05 tonnes of stainless steel for the sump and inner wall of the pumping station; the environmental impact of conventional steel is used (basic process #10). Dimensions and mass estimates derived from (Rostfria, 2017). | Cost as provided by the water department of Gällivare municipality (Eliasson, personal communication, December 13, 2016). Lifetime of 50 years is a common value for life cycle analysis of buildings. The amortization period is collected from (Nacka, 2012) and is typical for Swedish water utilities.  ↑ |
| Wastewater pumping station: Transport to construction site | 1 | - | 50 | 50 | ↑ | 7,02E+02 | 4,50E+01 | 2,74E-06 | Based on basic process #3 considering a mass of 3 tonnes^.^ Transportation over 300km from Storfors, Sweden. |  |
| Wastewater pump: Production | 1 | - | 25 | 25 | 2,00E+04 | 9,94E+02 | 1,11E+02 | -6,16E-04 | Based on basic process #10 considering 70kg of steel and on process #9 considering 2kg of copper wire. | Price, Lifetime and amortization period as proposed by (Nacka, 2012). |
| Wastewater pump: Operation and Maintenance | 1 | - | 1 | - | 5,80E+03 | 7,03E+03 | 5,12E+01 | 1,02E-04 | Lifting of wastewater from 100 residential units over 17 meters of elevation. Consumption of electricity (basic process #18) considering a pump efficiency of 55%. Environmental load of maintenance operations is neglected. | Cost corresponds to the average O&M expenditure for sewer pumping stations of Gällivare during 2013, 2014, 2015 divided by the number of pumping station in Gällivare (VASS, 2017). |
| Drinking water distribution network: O&M | 3710 | meter | 1 | - | 1,33E+00 | 6,44E+01 | 4,69E-01 | 9,37E-07 | Derived from O&M costs of drinking water distribution network considering that 50% of cost is due to electrical consumption (basic process #18). | Cost corresponds to the average O&M expenditure for the drinking water distribution network of Gällivare during 2013, 2014, 2015 divided by network length (VASS, 2017). |
| Heat pump for single family home : Production and installation of heat pump | 71 | - | 20 | 20 | 8,61E+03 | 2,50E+04 | 8,98E+02 | 9,03E-02 | Process 8.1.01 "Electric heat pump (brine-water, geothermal probe) 10 kW" from Oekobaudat 2016 database (BMUB, 2016). | Price of a NIBE F1255 3-12kW heat pump as found the 2017/12/08 on NIBE website. Price of installation according to (Bergvärmepumpar, 2018) is included. 25% VAT is excluded. Lifetime of 20 years is suggested by both the Oekobaudat 2016 database (BMUB, 2016) and the Danish energy agency (Energistyrelsen, 2012). Amortization period is taken equal to the lifetime. |
| Heat pump for single family home : Transport to construction site | 71 | - | 20 | 20 |  | 2,73E+02 | 1,75E+01 | 1,06E-06 | Based on basic process #3 considering 250kg/heat pump. Transportation over 1400km from NIBE facilities in Markaryd, Sweden. |  |
| Heat pump for single family home: Production and installation of boreholes and collectors | 71 | - | 50 | 25 | 3,52E+03 | 8,95E+03 | 5,78E+02 | 1,30E-04 | Process 8.1.01 "Pipework for electric heat pump (brine-water, geothermal probe) 10 kW" from Oekobaudat 2016 database (BMUB, 2016). | Price of material (pipework) and labour (drilling) is collected from (Bergvärmepumpar, 2018). 25% VAT is excluded. Lifetime of 50 years is suggested by (Annelie et al., 2012) on page 27. Amortization period of 20 years is suggested by the Swedish district heating association (SDHA, 2007). |
| Heat pump for single family home: Maintenance | 71 | - | 1 | - | 1,35E+02 | 0,00E+00 | 0,00E+00 | 0,00E+00 | Environmental load of heat pump maintenance is neglected. | Cost as estimated by the Danish Energy Agency (Energistyrelsen, 2012). |
| Heat pump for 20 apartments building : Production and installation of heat pump | 7 | - | 20 | 20 | 4,23E+04 | 3,15E+05 | 9,68E+03 | 3,75E-01 | Based on process 8.1.01 "Electric heat pump (brine-water, geothermal probe) 70 kW" from Oekobaudat 2016 database (BMUB, 2016) considering a capacity of 100kW. | Sum of prices of a NIBE F1345 60kw and NIBE F1345 40kw heat pump as found the 2017/12/08 on NIBE website. Includes the cost of installation as given by (Bergvärmepumpar, 2018) for a 10kw heat pump and multiplied by 10. 25% VAT is excluded. Lifetime of 20 years is suggested by both the Oekobaudat 2016 database (BMUB, 2016) and the Danish energy agency (Energistyrelsen, 2012). Amortization period is taken equal to the lifetime. |
| Heat pump for 20 apartments building : Transport to construction site | 7 | - | 20 | 20 | ↑ | 7,56E+02 | 4,84E+01 | 2,95E-06 | Based on basic process #3 considering 500kg/heat pump. Transportation over 1400km from NIBE facilities in Markaryd, Sweden. |  |
| Heat pump for 20 apartments building: Production and Installation of boreholes and collectors | 7 | - | 50 | 25 | 3,52E+04 | 8,95E+04 | 5,78E+03 | 1,30E-03 | Environmental load for a single family home is multiplied by 10 (proportional to capacity). | Price of material (pipework) and labour (drilling) described above for a single family home is multiplied by 10 (as 10 times more capacity is required). Lifetime of 50 years is suggested by (Annelie et al., 2012) on page 27. Amortization period of 20 years is suggested by the Swedish district heating association (SDHA, 2007). |
| Heat pump for 20 apartments building: maintenance | 7 | - | 1 | - | 4,00E+02 | 0,00E+00 | 0,00E+00 | 0,00E+00 | Environmental load of heat pump maintenance is neglected. | Cost as estimated by the Danish Energy Agency (Energistyrelsen, 2012). |
| Heat provided to single family home: Production with geothermal heat pump | 71 | - | 1 | - | 3,30E+02 | 3,32E+04 | 2,32E+02 | 4,65E-04 | Annual heat demand of 16900 kWh is considered. Swedish electricity is consumed (basic process #18) considering a coefficient of performance of 4. | Annual heat demand of 16900 kWh is considered. Electricity at 0,078/kWh (source:Vattenfall) is consumed considering a coefficient of performance of 4 for the heat pump. |
| Heat delivered to 20 apartments building: Production with geothermal heat pump | 7 | - | 1 | - | 3,14E+03 | 3,17E+05 | 2,21E+03 | 4,43E-03 | Annual heat demand of 161000 kWh is considered (20*8050kWh). Swedish electricity is consumed (basic process #18) considering a coefficient of performance of 4. | Annual heat demand of 161000 kWh is considered (20*8050kWh). Electricity at 0,078€/kWh (source: Vattenfall) is consumed considering a coefficient of performance of 4 for the heat pump. |

Table S6. Output flow, flow unit, lifetime, amortization period, cost and environmental impacts of the system processes composing alternative 5 (low pressure sewer and geothermal heat pumps).

| **System process** | **Output flow** | **Flow unit** | **Lifetime** | **Amortizati-on period** | **Cost** | **Cumulative Exergy Demand CExD** | **Global Warming Potential GWP** | **Abiotic Depletion Potential of Elements ADPE** | **Data sources and assumptions** | |
| --- | --- | --- | --- | --- | --- | --- | --- | --- | --- | --- |
|  |  |  | *Years* | *Years* | €*/unit* | *MJ/unit* | *KgCO_2_Eq/unit* | *KgSbEq/unit* | **Unitary environmental impacts (CExD, GWP, ADPE)** | **Unitary cost, lifetime and amortization period** |
| Trench for Low pressure sewer and drinking water pipe : Excavation with a 100 kW excavator | 3510 | meter | 60 | 50 | 7,00E+01 | 4,64E+00 | 3,12E-01 | 1,60E-08 | Based on basic process #1 considering 0,24 m^3^ of soil. | Cost of a shallow trench including installation of water and sewer pipes as provided by the water utility of Kiruna (Skoglind, 2014). Kiruna is a municipality nearby Gällivare with similar labour costs. Trench lifetime is limited to 60 years for the LPS sewer system due to the electric heating cable (Nacka, 2012). Amortization period is also obtained from (Nacka, 2012) and is typical for Swedish water utilities. |
| Trench for Low pressure sewer and drinking water pipe : Backfilling with a 100 kW excavator | 3510 | meter | 60 | 50 |  | 3,09E+00 | 2,08E-01 | 1,07E-08 | Based on basic process #1 considering 0,16 m^3^ of soil. |  |
| Crushed rock 0-8mm: Production | 3510 | meter | 60 | 50 |  | 6,35E+00 | 1,66E-01 | 3,33E-07 | Based on basic process #6 considering 0,15 m^3^ of 0-8mm crushed rocks. |  |
| Crushed rock 0-8mm: Transport to construction site | 3510 | meter | 60 | 50 |  | 3,43E+00 | 2,22E-01 | 1,35E-08 | Based on basic process #2 considering 0,15 m^3^ of 0-8mm crushed rocks at density 1400 kg/m^3.^ Transportation over 16km from LKAB Malmberget mine. |  |
| Remaining natural soil: Transport to disposal site | 3510 | meter | 60 | 50 |  | 1,88E+00 | 1,21E-01 | 7,40E-09 | Based on basic process #2 considering 0,23 m^3^ of natural soil at density 1600 kg/m^3.^ Transportation over 5km to disposal site. |  |
| XPS insulation boards: Installation | 3510 | meter | 60 | 50 |  | 0,00E+00 | 0,00E+00 | 0,00E+00 | Environmental load of XPS board installation is neglected. |  |
| LPS pipe in PE: Installation | 3510 | meter | 60 | 50 |  | 0,00E+00 | 0,00E+00 | 0,00E+00 | Environmental load of LPS pipe installation is neglected. |  |
| Drinking water pipes in PE: Installation | 3510 | meter | 60 | 50 |  | 0,00E+00 | 0,00E+00 | 0,00E+00 | Environmental load of the installation of the water pipes is neglected. |  |
| XPS insulation boards: Production | 3510 | meter | 60 | 50 | 3,35E+01 | 1,90E+02 | 6,72E+00 | 2,71E-06 | Based on basic process #13 considering 0,06m^3^ of XPS insulation. | Material costs as provided by the supplier “Skandinavisk Kommunalteknik AB” (T. Jansson, personal communication, November 17, 2016). Trench lifetime is limited to 60 years for the LPS sewer system due to the electric heating cable (Nacka, 2012). Amortization period is also obtained from (Nacka, 2012) and is typical for Swedish water utilities. |
| XPS insulation boards: Transport to construction site | 3510 | meter | 60 | 50 |  | 2,13E+00 | 1,37E-01 | 8,30E-09 | Based on basic process #3 considering 2,1kg of XPS insulation. Transportation over 1300km from Norrköping, Sweden. |  |
| Electric heating cable for freeze protection of water and sewer lines: Production | 3510 | meter | 60 | 50 |  | 3,94E-01 | 2,22E-02 | 7,88E-07 | Based on basic process #9 considering 0,03 kg of copper (wire diameter of 2mm and copper density 8960 kg/m^3^) |  |
| LPS pipes in PE: Production | 3510 | meter | 60 | 50 | ↑ | 8,07E+01 | 1,96E+00 | 3,52E-08 | Based on basic process #7 considering 1kg of PE. Only the production of raw material is considered as it represents most of the environmental load of PE pipes manufacturing (Carolin et al., 2011). | ↑ |
| LPS pipes in PE: Transport to construction site | 3510 | meter | 60 | 50 |  | 2,67E-01 | 1,72E-02 | 1,05E-09 | Based on basic process #2 considering 1kg of PE pipe. Transportation over 260km from Pipelife AB facilities in Haparanda, Sweden. |  |
| Drinking water pipes in PE: Production | 3510 | meter | 60 | 50 |  | 1,59E+02 | 3,88E+00 | 6,96E-08 | Based on basic process #7 considering 1,98kg of PE. Only the production of raw material is considered as it represents most of the environmental load of PE pipes manufacturing (Carolin et al., 2011). |  |
| Drinking water pipes in PE: Transport to construction site | 3510 | meter | 60 | 50 |  | 5,27E-01 | 3,41E-02 | 2,08E-09 | Based on basic process #2 considering 1,98kg of PE pipe. Transportation over 260km from Pipelife AB facilities in Haparanda, Sweden. |  |
| Electric heating cable for freeze protection of water and sewer lines: Operation | 3510 | meter | 1 | - | 6,64E-01 | 1,96E+02 | 1,43E+00 | 2,85E-06 | Use of 6W of electricity (basic process #18) during 6 months for freeze protection of water and sewer pipe. The heat tracing power of 6 W/m is estimated with the method proposed by (Gunderson, 1978). | Use of 6W of electricity at a price of 0,026 €/kWh (Vattenfall, 2017) during 6 months for freeze protection of water and sewer pipe. The heat tracing power of 6 W/m is estimated with the method proposed by (Gunderson, 1978). |
| Drinking water distribution network: O&M | 3510 | meter | 1 | - | 1,33E+00 | 6,44E+01 | 4,69E-01 | 9,37E-07 | Derived from O&M costs of drinking water distribution network considering that 50% of cost is due to electrical consumption (basic process #18). | Cost corresponds to the average O&M expenditure for the drinking water distribution network of Gällivare during 2013, 2014, 2015 divided by network length (VASS, 2017). |
| LPS tank for single family home : Production | 71 | - | 125 | 25 | 6,00E+02 | 5,61E+03 | 1,37E+02 | 2,45E-06 | Based on basic process #7 considering 70kg of PE. Only the production of raw material is considered. Weight of tank obtained directly from “Skandinavisk Kommunalteknik AB”, supplier of LPS solutions (T. Jansson, personal communication, November 17, 2016). | Costs as provided from “Skandinavisk Kommunalteknik AB”, supplier of LPS solutions (T. Jansson, personal communication, November 17, 2016). The same lifetime as for PE pipes in Sweden is used since the tanks are also made of PE and installed in the ground. Amortization period is obtained from (Nacka, 2012). |
| LPS tank for single family home : Transport to construction site | 71 | - | 125 | 25 |  | 5,84E+01 | 3,75E+00 | 2,28E-07 | Based on basic process #3 considering 70kg of PE. Transport over 1070km from Uppsala, Sweden. |  |
| LPS tank for single family home : Installation | 71 | - | 125 | 25 | 7,46E+02 | 1,45E+03 | 9,73E+01 | 4,99E-06 | Based on basic process #1 considering an excavation of 37,7m^3^ of sandy soil and backfilling of 36,9m^3^. Remaining soil (0,8m^3^) is transported by medium weight truck over 5 km to a disposal site (basic process #2). | Excavation of 37,7m^3^ of soil at 10€/m^3^ and backfilling of 36,9m^3^ of soil at 10€/m^3^. Excavation cost is provided by Gällivare municipality (Eliasson, personal communication, December 13, 2016). Lifetime and amortization period of the LPS tank are used. |
| LPS tanks for 20 apartments building : Production | 7 | - | 125 | 25 | 4,80E+03 | 1,99E+04 | 9,58E+02 | 7,29E-04 | 3 tanks of 180kg made of glass reinforced polyester (GRP). Production of raw material is modelled thought basic processes #15 #16 and #17 considering 500g of glass fibres, 320g of polyester resin and 160g of calcium carbonate per kg of GRP (Wang et al., 2013). | 1600€ per tank as provided by “Skandinavisk Kommunalteknik AB”, supplier of LPS solutions (T. Jansson, personal communication, November 17, 2016). Lifetime and amortization lifetime: same as LPS tank for single family home. |
| LPS tanks for 20 apartments building : Transport to construction site | 7 | - | 125 | 25 |  | 4,51E+02 | 2,89E+01 | 1,76E-06 | Based on basic process #3 considering 3*180kg of glass reinforced polyester. Transport over 1070km from Uppsala, Sweden. |  |
| LPS tanks for 20 apartments building : Installation | 7 | - | 125 | 25 | 2,77E+03 | 5,43E+03 | 3,65E+02 | 1,88E-05 | Based on basic process #1 considering an excavation of 143m^3^ of sandy soil and backfilling of 134m^3^. Remaining soil (9m^3^) is transported by medium weight truck over 5 km to a disposal site (basic process #2). | Excavation of 143m^3^ of soil at 10€/m^3^ and backfilling of 134m^3^ of soil at 10€/m^3^. . Excavation cost is provided by Gällivare municipality (Eliasson, personal communication, December 13, 2016). Lifetime and amortization period of the LPS tank are used. |
| LPS pump for single family home : Production | 71 | - | 25 | 25 | 1,70E+03 | 6,63E+02 | 7,26E+01 | -3,48E-04 | Based on basic process #10 and #9 considering 45kg of cast iron (environmental load of steel is used) and 3kg of copper wire. Mass estimates obtained from the supplier “Skandinavisk Kommunalteknik AB” (T. Jansson, personal communication, November 17, 2016). | Price as provided by “Skandinavisk Kommunalteknik AB”, supplier of LPS solutions (T. Jansson, personal communication, November 17, 2016). Lifetime and amortization period as suggested by Nacka municipality (Nacka, 2012). |
| LPS pump for single family home : Transport to construction site | 71 | - | 25 | 25 |  | 1,08E+02 | 6,48E+00 | 4,22E-07 | Transport of the 48kg pump over 6000km by sea cargo (basic process #3) from New York to Gothenburg. Transport over 1500 km by heavy truck (basic process #4) from Gothenburg to Gällivare. |  |
| LPS pump for single family home : Installation | 71 | - | 25 | 25 |  | 0,00E+00 | 0,00E+00 | 0,00E+00 | The environmental load of heat pump installation is neglected. |  |
| LPS pump for single family home : Operation | 71 | - | 1 | - | 3,91E+00 | 3,78E+02 | 2,75E+00 | 5,50E-06 | Use of 50 kWh per year of electricity (basic process #18) as provided by (Lindqvist et al., 2000). | Use of 50 kWh per year of electricity as provided by (Lindqvist et al., 2000). Electricity price of 0,078 €/kWh (source:Vattenfall) is used. |
| LPS pump for single family home : Maintenance | 71 | - | 1 | - | 1,50E+01 | 0,00E+00 | 0,00E+00 | 0,00E+00 | The environmental load of pump maintenance is neglected. | Maintenance operation needs to be done in average 1,5 times during the 25 years life cycle of the pump and costs 250€. Data communicated by the supplier “Skandinavisk Kommunalteknik AB” (T. Jansson, personal communication, November 17, 2016). |
| LPS pumps for 20 apartments building : Production | 7 | - | 25 | 25 | 1,70E+04 | 6,63E+03 | 7,26E+02 | -3,48E-03 | Based on basic process #10 and #9 considering 10*45kg of cast iron (environmental load of steel is used) and 10*3kg of copper wire. Mass estimates obtained from the supplier “Skandinavisk Kommunalteknik AB” (T. Jansson, personal communication, November 17, 2016). | 10 pumps with unitary cost 1700€ as provided by “Skandinavisk Kommunalteknik AB”, supplier of LPS solutions (T. Jansson, personal communication, November 17, 2016). Lifetime and amortization period as suggested by Nacka municipality (Nacka, 2012). |
| LPS pumps for 20 apartments building : Transport to construction site | 7 | - | 25 | 25 |  | 1,08E+03 | 6,48E+01 | 4,22E-06 | Transport of 10 pumps (10*48kg) over 6000km by sea cargo (basic process #3) from New York to Gothenburg. Transport over 1500 km by heavy truck (basic process #4) from Gothenburg to Gällivare. |  |
| LPS pumps for 20 apartments building : Installation | 7 | - | 25 | 25 |  | 0,00E+00 | 0,00E+00 | 0,00E+00 | The environmental load of pump installation is neglected. |  |
| LPS pumps for 20 apartments building : Operation | 7 | - | 1 | - | 7,81E+01 | 7,56E+03 | 5,50E+01 | 1,10E-04 | Use of 20*50 kWh per year of electricity (basic process #18). | Use of 20*50 kWh per year of electricity at a price of 0,078 €/kWh (source:Vattenfall). |
| LPS pumps for 20 apartments building : Maintenance | 7 | - | 1 | - | 1,50E+02 | 0,00E+00 | 0,00E+00 | 0,00E+00 | The environmental load of pump maintenance is neglected. | Maintenance operation needs to be done in average 1,5 times during the 25 years life cycle of each pump and costs 250€. 10 pumps are used for a 20 apartment building. Data communicated by “Skandinavisk Kommunalteknik AB” (T. Jansson, personal communication, November 17, 2016). |
| Heat pump for single family home : Production and installation of heat pump | 71 | - | 20 | 20 | 8,61E+03 | 2,50E+04 | 8,98E+02 | 9,03E-02 | Process 8.1.01 "Electric heat pump (brine-water, geothermal probe) 10 kW" from Oekobaudat 2016 database (BMUB, 2016). | Price of a NIBE F1255 3-12kW heat pump as found the 2017/12/08 on NIBE website. Price of installation according to (Bergvärmepumpar, 2018) is included. 25% VAT is excluded. Lifetime of 20 years is suggested by both the Oekobaudat 2016 database (BMUB, 2016) and the Danish energy agency (Energistyrelsen, 2012). Amortization period is taken equal to the lifetime. |
| Heat pump for single family home : Transport to construction site | 71 | - | 20 | 20 |  | 2,73E+02 | 1,75E+01 | 1,06E-06 | Based on basic process #3 considering 250kg/heat pump. Transportation over 1400km from NIBE facilities in Markaryd, Sweden. |  |
| Heat pump for single family home: Production and installation of boreholes and collectors | 71 | - | 50 | 25 | 3,52E+03 | 8,95E+03 | 5,78E+02 | 1,30E-04 | Process 8.1.01 "Pipework for electric heat pump (brine-water, geothermal probe) 10 kW" from Oekobaudat 2016 database (BMUB, 2016). | Price of material (pipework) and labour (drilling) is collected from (Bergvärmepumpar, 2018). 25% VAT is excluded. Lifetime of 50 years is suggested by (Annelie et al., 2012) on page 27. Amortization period of 20 years is suggested by the Swedish district heating association (SDHA, 2007). |
| Heat pump for single family home: Maintenance | 71 | - | 1 | - | 1,35E+02 | 0,00E+00 | 0,00E+00 | 0,00E+00 | Environmental load of heat pump maintenance is neglected. | Cost as estimated by the Danish Energy Agency (Energistyrelsen, 2012). |
| Heat pump for 20 apartments building : Production and installation of heat pump | 7 | - | 20 | 20 | 4,23E+04 | 3,15E+05 | 9,68E+03 | 3,75E-01 | Based on process 8.1.01 "Electric heat pump (brine-water, geothermal probe) 70 kW" from Oekobaudat 2016 database (BMUB, 2016) considering a capacity of 100kW. | Sum of prices of a NIBE F1345 60kw and NIBE F1345 40kw heat pump as found the 2017/12/08 on NIBE website. Includes the cost of installation as given by (Bergvärmepumpar, 2018) for a 10kw heat pump and multiplied by 10. 25% VAT is excluded. Lifetime of 20 years is suggested by both the Oekobaudat 2016 database (BMUB, 2016) and the Danish energy agency (Energistyrelsen, 2012). Amortization period is taken equal to the lifetime. |
| Heat pump for 20 apartments building : Transport to construction site | 7 | - | 20 | 20 |  | 7,56E+02 | 4,84E+01 | 2,95E-06 | Based on basic process #3 considering 500kg/heat pump. Transportation over 1400km from NIBE facilities in Markaryd, Sweden. |  |
| Heat pump for 20 apartments building: Production and Installation of boreholes and collectors | 7 | - | 50 | 25 | 3,52E+04 | 8,95E+04 | 5,78E+03 | 1,30E-03 | Environmental load for a single family home is multiplied by 10 (proportional to capacity). | Price of material (pipework) and labour (drilling) described above for a single family home is multiplied by 10 (as 10 times more capacity is required). Lifetime of 50 years is suggested by (Annelie et al., 2012) on page 27. Amortization period of 20 years is suggested by the Swedish district heating association (SDHA, 2007). |
| Heat pump for 20 apartments building: Maintenance | 7 | - | 1 | - | 4,00E+02 | 0,00E+00 | 0,00E+00 | 0,00E+00 | Environmental load of heat pump maintenance is neglected. | Cost as estimated by the Danish Energy Agency (Energistyrelsen, 2012). |
| Heat provided to single family home: Production with geothermal heat pump | 71 | - | 1 | - | 3,30E+02 | 3,32E+04 | 2,32E+02 | 4,65E-04 | Annual heat demand of 16900 kWh is considered. Swedish electricity is consumed (basic process #18) considering a coefficient of performance of 4. | Annual heat demand of 16900 kWh is considered. Electricity at 0,078/kWh (source:Vattenfall) is consumed considering a coefficient of performance of 4 for the heat pump. |
| Heat delivered to 20 apartments building: Production with geothermal heat pump | 7 | - | 1 | - | 3,14E+03 | 3,17E+05 | 2,21E+03 | 4,43E-03 | Annual heat demand of 161000 kWh is considered (20*8050kWh). Swedish electricity is consumed (basic process #18) considering a coefficient of performance of 4. | Annual heat demand of 161000 kWh is considered (20*8050kWh). Electricity at 0,078€/kWh (source: Vattenfall) is consumed considering a coefficient of performance of 4 for the heat pump. |

**References**

Annelie, K., Quistgaar, L., Jardeby, Å., Räftegår, O., 2012. Boreholes and aquifers (Borrhåls- och grundvattenlager, in Swedish). Retrieved 05/02/18 from Swedish Technical Research Institute (SP) website: https://www.sp.se/sv/index/research/eu-project/interreg/geopower/Documents/Borrhals och grundvattenlager_praktisk handbok om geoenergi.pdf

Bergvärmepumpar, 2018. Price and cost for geothermal heat pumps (Pris & kostnad för bergvärmepumpar, in Swedish). http://www.bergvarmepumpar.n.nu/bergvarmepump-pris (accessed 2/2/18).

BMUB, 2016. ÖKOBAUDAT platform by the German Ministry for the Environment, Nature Conservation, Building and Nuclear Safety. http://www.oekobaudat.de/en.html (accessed 29/10/17).

Bösch, M.E., Hellweg, S., Huijbregts, M.A.J., Frischknecht, R., 2007. Applying cumulative exergy demand (CExD) indicators to the ecoinvent database. Int. J. Life Cycle Assess. 12, 181–190. doi:10.1065/lca2006.11.282

Carolin, S., Vanderreydt, I., Vercalsteren, A., Boonen, K., 2011. Life Cycle Assessment of a PE pipe system for water distribution (according to EN 12201). VITO NV, Brussel, Belgium.

Carolin, S., Vanderreydt, I., Vercalsteren, A., Boonen, K., Peeters, K., 2012. Life Cycle Assessment of a PVC-U solid wall sewer pipe system (according to EN 1401). VITO NV, Brussel, Belgium.

ECJRC, 2012. European Reference Life Cycle Database (ELCD). Version 3.2. European Commission - Joint Research Centre - Institute for Environment and Sustainability. http://eplca.jrc.ec.europa.eu/ELCD3/ (accessed 29/10/17).

Energistyrelsen, 2012. Technology data for energy plants. Retrieved 05/02/2018 from Danish Energy Agency webiste: https://ens.dk/sites/ens.dk/files/Analyser/c_teknologikatalog_for_individuelle_varmeanlaeg_og_energitransport_2012.pdf.

Erlandsson, M., 2010. Environmental data of aggregates and gravel (Miljödata för krossprodukter och naturgrus, in Swedish). Retrieved 05/02/18 from Swedish Environmental Research Institute webiste: http://www.ivl.se/download/18.343dc99d14e8bb0f58b76e6/1449742446674/C12.p.

Frederiksen, S., Werner, S., 2013. District Heating and Cooling. Studentlitteratur AB.

GEAB, 2016. Operation and business report 2015 for district heating (2015: DoA Fjärrvärme, in Swedish), Gällivare energi AB. Retrieved 05/02/18 from Swedish Energy Markets Inspectorate website: https://www.ei.se/sv/start-fjarrvarmekollen/foretag/gallivare-energi-ab/?Rapport=2016-101256.pdf

Gunderson, P., 1978. Frost Protection of Buried Water and Sewage Pipes (draft translation 666). Cold Region Research and Engineering Laboratory, Hanover, NH, Cold Region Engineering Laboratory report: draft translation 666.

Hagberg, L., Särnholm, E., Gode, J., Ekvall, T., Rydberg, T., 2009. LCA calculations on Swedish wood pellet production chains. Retrieved 05/02/18 from Swedish Environmental Institute website: http://www.ivl.se/download/18.343dc99d14e8bb0f58b7597/1445517411880/B1873.pdf.

IBU, 2014a. Environmental Product Declaration EPD-PUE-20130286-CBE1-EN: PU thermal insulation board with mineral fleece facing. Institut Bauen und Umwelt e.V., Berlin, Germany.

IBU, 2014b. Environmental Product Declaration EPD-EXI-20140154-IBE1-EN: Extruded Polystyrene (XPS) Foam Insulation with HBCD flame retardant. Institut Bauen und Umwelt e.V., Berlin, Germany.

IBU, 2013. Environmental Product Declaration EPD-EUM-20160272-IBG1-EN: Expanded Polystyrene (EPS) Foam Insulation. Institut Bauen und Umwelt e.V., Berlin, Germany.

Lindqvist, B.G., Lönnbring, J., Persson, G., Svensson, H., 2000. Low Pressure Sewerage Systems with lesser dimater piping (Tryckavloppssystem av rör med klena dimensioner, in Swedish). Swedish Water and Wastewater Association, Bromma, Sweden.

Malm, A., Svensson, G., Bäckman, H., Morrison, G.M., 2013. Prediction of water and wastewater networks rehabilitation based current age and material distribution. Water Sci. Technol. Water Supply 13, 227–237. doi:10.2166/ws.2013.011

Nacka, 2012. Comparative study between LPS and gravity sewer system (Jämförelsestudie LTA-Självfallssystem, in Swedish). Nacka Municipality, Nacka, Sweden.

Patel, M., 2003. Cumulative energy demand (CED) and cumulative CO2 emissions for products of the organic chemical industry. Energy 28, 721–740. doi:10.1016/S0360-5442(02)00166-4

Perzon, M., Johansson, K., Fröling, M., 2007. Life cycle assessment of district heat distribution in suburban areas using PEX pipes insulated with expanded polystyrene. Int. J. Life Cycle Assess. 12, 317–327. doi:10.1065/lca2006.08.264

Powerpipe, 2017. English Catalogue. Retrieved 05/02/18 from: http://www.powerpipe.se/en/products/english-catalogue.

Rostfria, 2017. Quick facts on stainless sewage pumping stations (Snabbfakta för våra rostfriapumpstationer, in Swedish). Rostfria VA-System i Storfors AB. http://rostfriapumpstationer.se/fakta/ (accessed 12/11/17).

SDHA, 2015. Maintenance handbook for district heat distribution developped by the Swedish District Heating Association (Underhållshandboken för fjärrvärmedistribution framtagen av svensk fjärrvärme, in Swedish). Retrieved 05/02/18 from: http://www.svenskenergi.se/Global/Dokument/publikationer/Underhallshandboken_2015.pdf

SDHA, 2007. Analysis of cost data for residential heating alternatives (Analys av Uppvärmningsalternativens Kostnadsposter, in Swedish). Swedish District Heating Association, Stockholm, Sweden.

SIS, 2003. Swedish standard SS-EN 253 District heating pipes - Preinsulated bonded pipe systems for directly buried hot water networks - Pipe assembly of steel service pipe, polyurethane thermal insulation and outer casing of polyethylene. Swedish Standards Institut.

Skoglind, A., 2014. Interview of A. Skoglind (TVAB i Kiruna) by T. Vikström (Luleå University of Technology) the 1/12/2014.

SLCC, 2000. CPM LCA database by Swedish Life Cycle Center, http://cpmdatabase.cpm.chalmers.se/ (accessed 2.15.17).

Vaisanen, S.E., Silvan, N.R., Ihalainen, A.V.J., Soukka, R.M., 2013. Peat Production in High-Emission Level Peatlands - a Key To Reducing Climatic Impacts? Energy Environ. 24, 757–778. doi:10.1260/0958-305X.24.5.757

VASS, 2017. Swedish Water and Wastewater Association Statistic System (Svenskt Vattens Statistik System, in Swedish). http://www.vass-statistik.se/ (accessed 15/12/17).

Vattenfall, 2017. Electrical subscriptions 2017 area north (Säkringsabonnemang 2017 område norr, in Swedish). Retrieved 05/02/18 from: https://www.vattenfalleldistribution.se/globalassets/el-hem-till-dig/elnatspriser/prislistor-2017/sakrings-norr-2017.pdf

Wang, J., Shi, S.Q., Liang, K., 2013. Comparative Life-cycle Assessment of Sheet Molding Compound Reinforced by Natural Fiber vs. Glass Fiber. J. Agric. Sci. Technol. B3, 493–502.

Yarahmadi, N., Sällström, J.H., 2014. Annex X Final Report: Improved maintenance of DH-pipes. IEA-DHC-CHP project. 05/02/18 from: https://www.kdhc.co.kr/contentDown.do?sgrup=S10&cmsCd=CM4255&fNo=10_01.pdf

Åkerström, Å., 2004. Re-investment model for existing district heating networks (Reinvesteringsmodell för befintligt fjärrvärmenät, in Swedish). Thesis project, Technical Faculty of Lund University (LTH).
